# Online media reveals a global problem of discarded containers as deadly traps for animals

**DOI:** 10.1038/s41598-020-79549-8

**Published:** 2021-01-11

**Authors:** Krzysztof Kolenda, Monika Pawlik, Natalia Kuśmierek, Adrian Smolis, Marcin Kadej

**Affiliations:** 1 Amphibian Biology Group, Department of Evolutionary Biology and Conservation of Vertebrates, Institute of Environmental Biology, University of Wrocław, Sienkiewicza 21, 50-335 Wrocław, Poland; 2 Department of Invertebrate Biology, Evolution and Conservation, Institute of Environmental Biology, University of Wrocław, Przybyszewskiego 65, 51-148 Wrocław, Poland; 3 Department of Parasitology, Institute of Genetics and Microbiology, University of Wrocław, Przybyszewskiego 63, 51-148 Wrocław, Poland

**Keywords:** Ecology, Biodiversity, Conservation biology

## Abstract

The widespread occurrence of litter is a severe threat to global ecosystems. We have analyzed online media, to assess the diversity of animals that are prone to getting trapped in discarded containers and check which kind of containers is the most common trap for animals. A total of 503 records from around the world (51 countries, 6 continents) have been found. These include invertebrates (17 taxa, ca.1050 dead individuals), and vertebrates (98 taxa, 496 individuals including 44 carcasses). The latter group was most frequently represented by mammals (78.5% of all cases), then reptiles (15.3%), birds (1.2%), fish (1.0%) and amphibians (0.4%). Nearly 12.5% of the determined vertebrates are classified as vulnerable, endangered or critically endangered, according to the IUCN. Although most trapped individuals were smaller animals, bigger ones such as monitor lizards (*Varanus* spp.) or large carnivores were also recorded. In most cases, animals were trapped in glass or plastic jars (32.4%), drink cans (16.5%), and steel cans (16.3%). Our results demonstrate that discarded containers can be a threat to all major groups of animals. In order to address this phenomenon, it is necessary to decrease a global production of debris, implement container deposit legislation and organize repeatable cleanup actions.

## Introduction

Growing human population and urbanization has led to a decrease in natural habitats and thus to human-wildlife conflicts^1^. Currently, one of the major environmental challenges is litter pollution^2,3^. In 2016, more than two billion tons of debris were produced worldwide^3^. Landfilled sites poses a serious threat to ecosystems, e.g. by penetration of toxic substances into the soil^4^ and water^5^, or the release of greenhouse gases^6^. Over time, litter that accumulates in the environment becomes a permanent element of ecosystems or even creates new ecosystems like the Great Pacific Garbage Patch^7^. Animals get used to the presence of litter and some groups even prefer littered habitats^8^ and use anthropogenic particles for nest building^9^ or to nest inside^10,11^. Another problem is that some litter becomes evolutionary traps for animals which confuse them with mates or food^12^. Anthropogenic products are ingested by both terrestrial and aquatic fauna^13–15^. Animals can ingest directly (when consuming prey or attacking items resembling prey) or indirectly (by ingesting prey which itself contains debris) small particles such as micro- or macro-plastic^16,17^, but also bigger items such as single-use plastic bags, bottles, ropes, and fishing lines which commonly cause internal injuries or death^13,18,19^.

Discarded food and beverage containers are currently one of the most common litter categories in the environment^20^ and offer a specific kind of ecological trap. The characteristic smell of putrefaction attracts many animals, which may suffer injury or get trapped when trying to extract food remains^21–24^. Animals dying in this way can also be “bait” for other, e.g. necrophagous organisms. Additionally, some rodents use discarded containers as shelter or enter containers during exploratory activities^25–27^. Consequently, a vast number of containers constitute a lethal trap for animals. Despite reports suggesting that invertebrates are the most threatened^28–30^, most authors have focused on the effect of litter traps on small mammals^23,25,26,31^ and there are only single reports mentioning mortality of amphibians or reptiles^22,23,32^. However, to our knowledge there are no reports suggesting the potential risk of discarded containers for birds and larger mammals such as ungulates or carnivores.

In recent years, scientists are increasingly embracing Internet resources, especially when data from the literature appear to be outdated or insufficient^33,34^. Reasons for this trend are the dynamic development of technology, easy access to the Internet, and a resulting increased flow of information^35,36^. Alternative data sources, other than conventional scientific literature, can provide completely new information or supplement existing knowledge^33,37^. In particular, it applies to social networks like YouTube, Twitter, Instagram or Facebook, which allow the collection of large-scale data from various fields of science^34^. Moreover, it is free and relatively quick to obtain the source of data that would often be difficult to receive by traditional research methods. Data obtained from the Internet have been used for many years and in many fields of science, especially social sciences such as psychology, sociology or human behavior^38–40^. They are also increasingly used in natural sciences, including animal behavior^37,41^, environmental protection^42^, wildlife monitoring^43^, human-animal and/or nature interaction^44–46^, and conservation biology^47–49^.

Because the knowledge of the impact of discarded containers on fauna is still insufficient, the aims of the current study were to assess: (i) the diversity of animals that are prone to getting trapped in discarded containers by using data shared on online media by citizens, (ii) which type of containers form the most common trap for animals.

## Results

**Figure 1:**
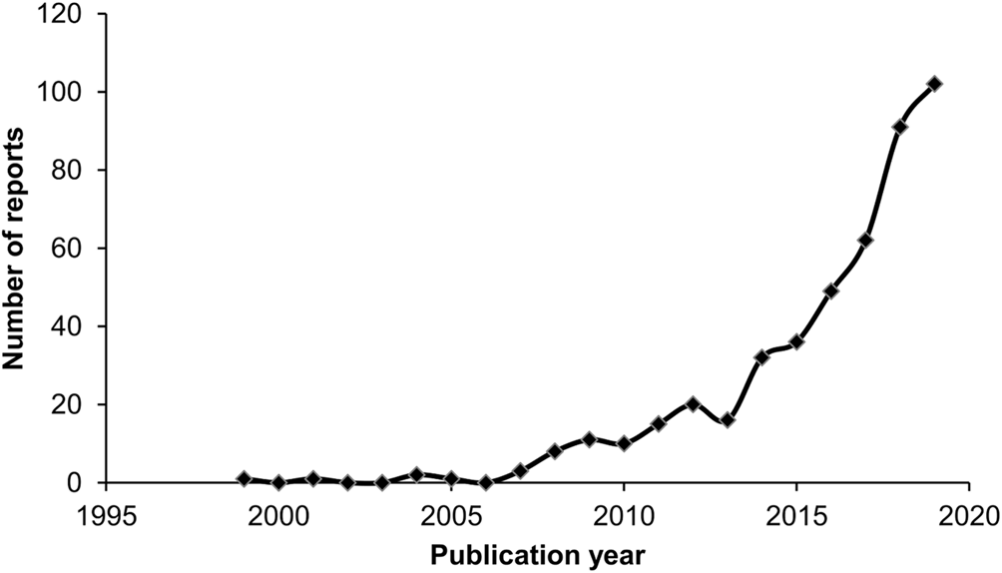
Number of reports concerning animals getting stuck in discarded containers shared on social media between July 1999–November 2019.

A total of 491 reports (184 movies, 307 photos) of 503 containers where animals got stuck inside were collected (see Supplementary Table S1 online). The data included events published between July 1999 and November 2019. Spearman's rank correlation showed a significant positive association between the year of publication and the number of reports (Spearman r = 0.95, p = 0.0001; Fig. 1). In 456 (90.6%) cases, location was allocated across a total of 51 countries and 6 continents (see Supplementary Table S2 online). Six cases (1.2%) could be classified only at continental level and in 41 remaining cases (8.2%) the location could not be determined. Most events occurred in the USA (176), Great Britain (46), followed by Australia and India (42 each).

In 386 cases, it was possible to classify the habitat where the container with the trapped animal was found. We found that these were most often urbanized habitats (n = 278 reports, 72%) rather than natural/semi-natural (n = 108, 28%) (chi-square test = 149.7, df = 1, p < 0.0001).

**Figure 2:**
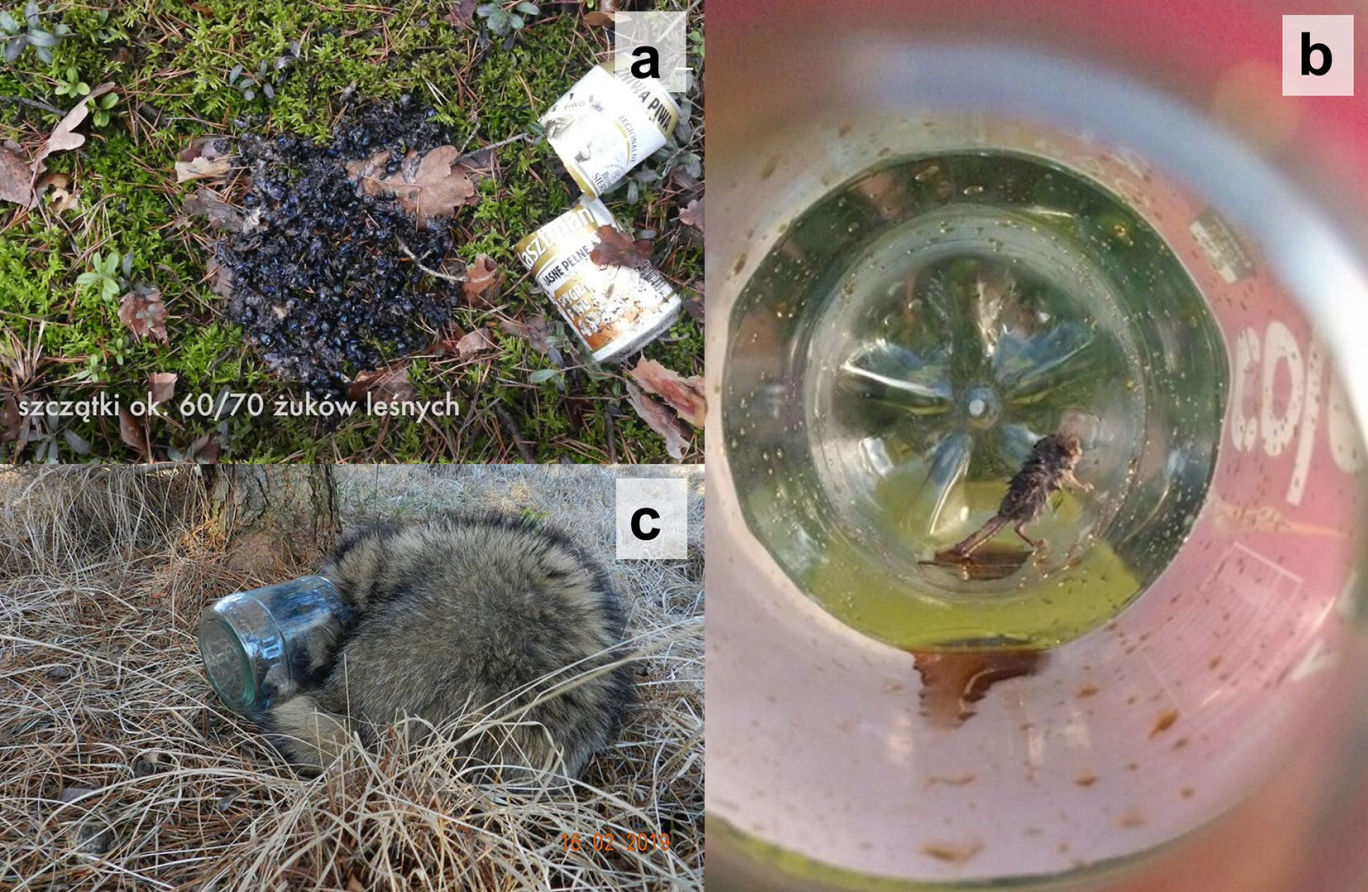
Examples of animals that got stuck in different type of discarded containers: (**a**) dor beetle *Anoplotrupes stercorosus* in beer can, translation of the caption in the photo: remains of ca. 60–70 dor beetles, photo by Konrad Hozler, source: https://www.youtube.com/watch?v=7o5aisetJDY, (**b**) harvest mouse *Micromys minutus* in Coca-Cola plastic bottle, photo by Olga Wojciechowska, source: https://www.facebook.com/photo.php?fbid=889881771382671&set=p.889881771382671&type=3&theater, (**c**) racoon dog *Nyctereutes procyonoides* in glass jar, photo by Bartosz Jaszewski, source: https://www.facebook.com/photo.php?fbid=2058677897551258&set=pcb.2058724980879883&type=3&theater&ifg=1.

Invertebrates (Fig. 2a) were found in 20 (4%) containers (10 drink cans, 9 bottles, and 1 cup). Among them, 17 taxa belonging to five major taxonomic units (Arachnida, Diplopoda, Gastropoda, Insecta, Malacostraca) were distinguished, of which 8 were successfully determined to a species level (see Supplementary Table S3 online). Except for two crabs and one fly, all invertebrates were found dead. In total, about 1050 individuals died in containers.

Most containers (n = 485, 96.4% of all) constituted a trap for a total of 496 vertebrates (Fig. 2b,c), which were classified into 98 taxa, including 80 to species level (see Supplementary Table S4 online). The most numerous order represented was mammals, which got stuck in 395 (78.5%) containers, followed by reptiles (n = 77, 15.3%), birds (n = 6, 1.2%), fish (n = 5, 1.0%) and amphibians (n = 2, 0.4%). Three individuals of single species were found trapped in four separate containers; the species were viviparous lizard *Zootoca vivipara*, southern alligator lizard *Elgaria multicarinata*, wood mouse *Apodemus sylvaticus*, and Trowbridge's shrew *Sorex trowbridgii*. Two goats *Capra hircus* had their heads stuck in the same item of litter. In addition, two individuals of different taxa (cat *Felis catus* with an undetermined mouse, and two species of lizards: sand lizard *Lacerta agilis* with viviparous lizard) were found in single containers on two separate occasions. In the remaining cases, containers contained only a single vertebrate. Additionally, in two containers with vertebrates, invertebrates were found as well.

Usually, the vertebrate's head was stuck (n = 447, 92.1%) in a container. Whole body entrapment was noted less frequently (n = 30, 6.1%) and, in the case of only 9 (1.8%) animals, it was a body part such as a tongue or paw.

In five (1.0%) cases, vertebrates were able to escape themselves from the containers. In 394 (81.2%), they were rescued by a human and, in 9 (1.9%) cases an unsuccessful attempt was made to help (an animal, partially stuck in a container, ran away). In 36 (7.4%) containers, the animals found were dead (a total of 44 dead individuals), and in three cases (0.6%) they had to be euthanized after being found. In the remaining 38 (7.9%) cases, no information was found regarding whether animals were able to free themselves or if someone helped them. Animals that were rescued from 144 containers were injured (most often: open wounds, swelling, dehydratation, and breathing problems).

Among 80 identified vertebrate species, 72 are listed on the IUCN Red List, including 63 (87.5%) species in the least concern (LC) category, followed by vulnerable (VU; n = 6, 8.3%), endangered (EN; n = 2, 2.8%), and critically endangered (CR; n = 1, 1.4%) (see Supplementary Table S4 online). The proportion of species in the LC category significantly differed from the number of species considered to be endangered (VU, EN and CR classified together; chi-square test = 81, df = 1, p < 0.0001).

A glass or plastic jars were the containers in which trapped animals were the most often observed (163 cases, 32.4%), followed by drink cans (n = 83, 16.5%), steel cans (n = 82, 16.3%), cups (n = 58, 11.5%) and bottles (n = 57, 11.4%). The remaining 60 (11.9%) were classified as ‘others’ (Table 1).

**Table 1:** Percentage share of particular group of animals that got stuck in different types of discarded containers.

	Bottle		Jar		Drink can		Steel can		Cup		Others	
	nc	%	nc	%	nc	%	nc	%	nc	%	nc	%
Invertebrates	9	15.5	0	0	10	11.9	0	0	1	1.7	0	0
Fishes	4	6.9	0	0	0	0	1	1.2	0	0	0	0
Amphibians	0	0	1	0.6	1	1.2	0	0	0	0	0	0
Reptiles	8	13.8	1	0.6	58	69	6	7.3	2	3.4	2	3.3
Birds	2	3.4	0	0	3	3.6	1	1.2	0	0	0	0
Mammals	35	60.3	161	98.8	12	14.3	74	90.2	55	94.8	58	96.7
**Reptiles**												
Lizards	6	75	0	0	15	25.9	5	83.3	0	0	1	50
Snakes	2	25	1	100	43	74.1	1	16.7	0	0	0	0
Turtles	0	0	0	0	0	0	0	0	2	100	1	50
**Mammals**												
Small	14	40	5	3.1	3	25	4	5.4	29	52.7	3	5.3
Medium	21	60	148	91.4	9	75	68	91.9	26	47.3	35	61.4
Large	0	0	9	5.6	0	0	2	2.7	0	0	19	33.3

*NC* number of containers.

**Figure 3:**
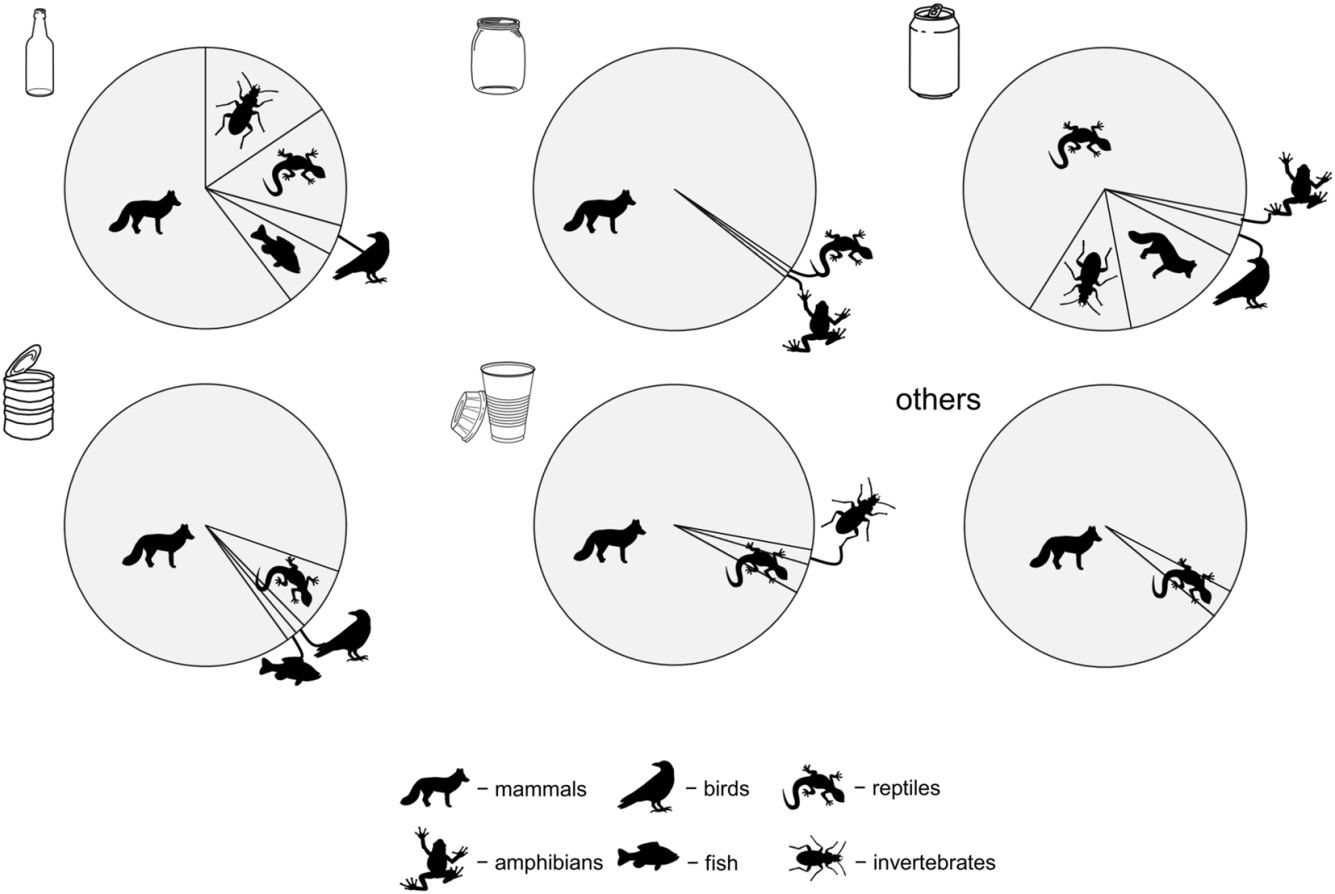
Share of particular animals that got stuck in different types of discarded containers. Container symbols mean (from upper left corner): bottle, jar, drink can, steel can, cup.

The most common order in almost all container types was mammals; in jars, cups, and ‘others’ they constituted more than 90% of all described cases (Table 1, Fig. 3). Moreover, they accounted for over 60% of all bottle victims. The only exception were drink cans, were reptiles dominated (69%). The proportion of particular orders of vertebrates significantly differed between container types (chi-square test = 340.1, df = 25, p < 0.0001; Table 1).

**Figure 4:**
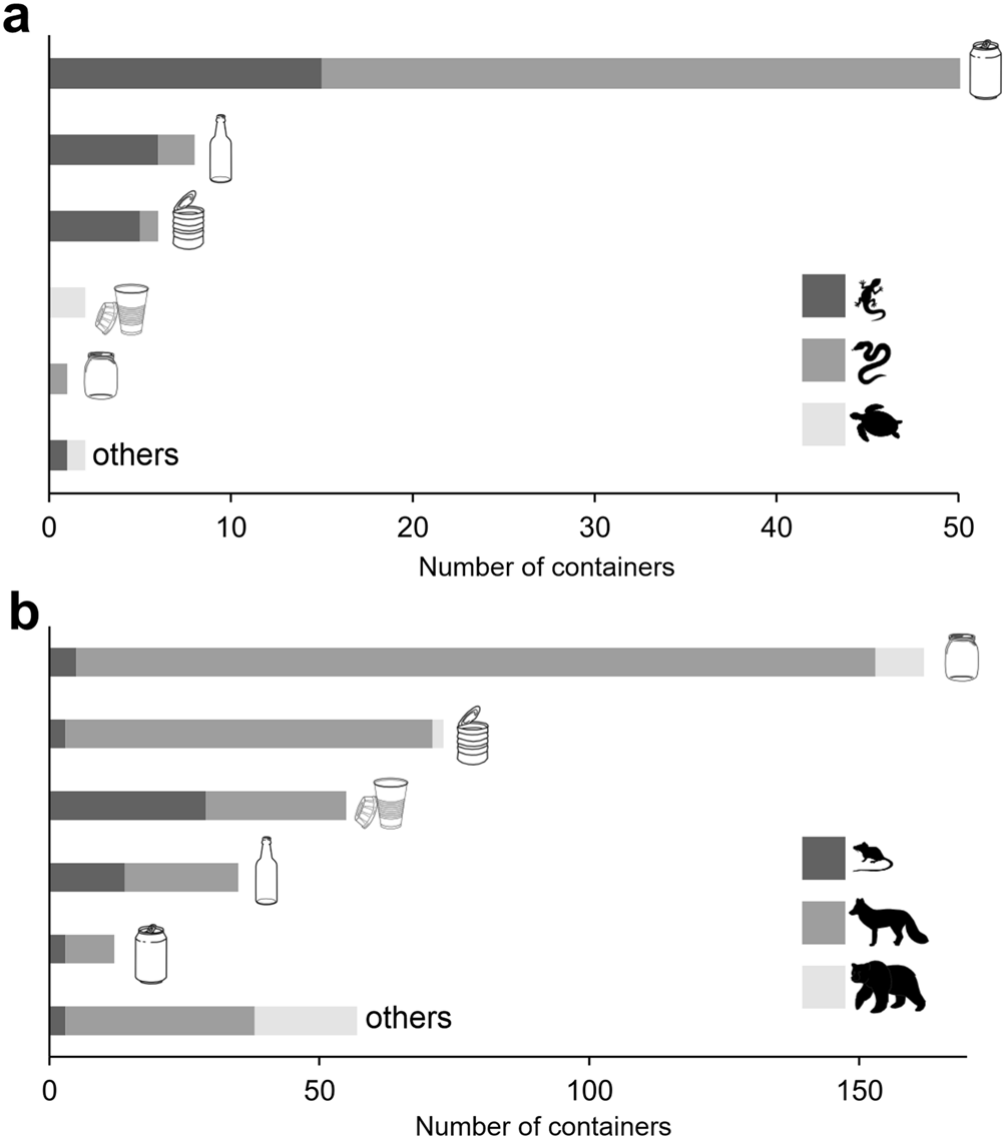
Number of reptiles (**a**) and mammals (**b**) that got stuck in different types of discarded containers. Animal symbols mean: (**a**) lizards, snakes, turtles, and (**b**) small, medium and large size mammals, respectively. Container symbols as in Fig. 3.

**Figure 5:**
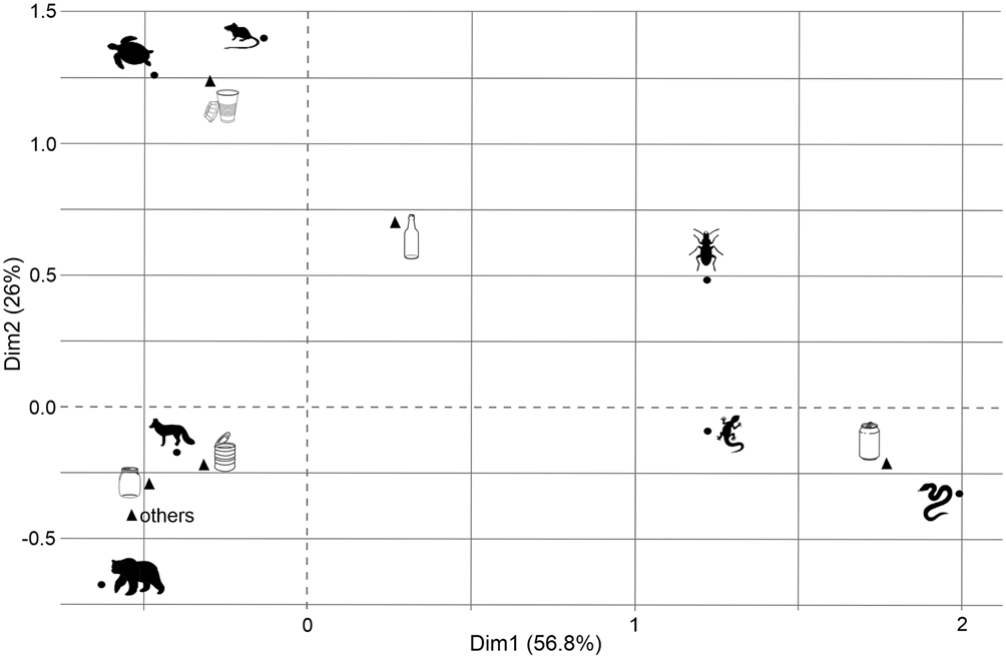
Correspondence analysis showing relationships between the most common groups of animals: invertebrates, reptiles (lizards, snakes, turtles), mammals (small, medium, large) and different types of discarded containers (symbols as in Fig. 3).

Among reptiles, drink cans constituted the highest threat to snakes (43 containers, 74.1% of all with reptiles), while bottles and steel cans to lizards (n = 6, 75% and n = 5, 83.3%, respectively). Furthermore, only turtles got stuck in cups, and one snake was trapped in a jar (Table 1, Figs. 4a, 5).

Small mammals dominated in cups (n = 29, 52.7% of all with mammals), while ‘other’ types of container were most dangerous to medium size mammals. ‘Other’ types of container also posed a relatively high threat to large mammals (n = 19, 33.3%) (Table 1, Figs. 4b, 5). The proportion of a particular group of mammals significantly differed between the types of container (chi-square test = 156.9, df = 10, p < 0.0001).

## Discussion

Data collected during the course of the study came from six continents, covered various habitats—from cities to undisturbed wilderness—and concerned both invertebrates and vertebrates. This indicates that the impact of littering in the environment on fauna is a widespread global problem. It should be emphasized that, due to the specific selection of keywords used to search and probable inaccurate translation of some of them by Google Translate, the data collected during this study only covers a proportion of the data available on the Internet. In addition, undoubtedly not all cases that occurred in the past were noticed, documented, and made public, especially those in which the whole animal has been trapped in a container, as these could be easily overlooked. This is confirmed by the results of this study: only 4% of the containers contained dead invertebrates, and only 5.9% of the containers with vertebrates concerned small animals with the whole body stuck. It is also possible that some of the container victims were eaten by scavengers^23^. Similar problems can be encountered during the estimation of the scale of animal mortality on roads^50^. The abovementioned factors therefore generate problems in estimating the real scale of the problem under study; however, based on few literature sources, it can be assumed that the extent of this phenomenon is huge. Moates^31^ suggested that 2.9 million small mammals such as mice, shrews, and voles die in discarded containers in Great Britain each year, while Lavers et al.^30^ estimate a combined mortality of half a million hermit crabs on the beaches of the Cocos and Henderson Islands.

The available data indicates a predominant share of invertebrates and small mammals (mice, shrews, voles) among animals threatened with getting stuck in discarded containers^21,23–31^. The main cause of this phenomenon may be the widespread occurrence of these animals in the environment at high densities and, because they are small, they can easily enter any type of container. However, this is not supported by our results, because most cases shared on the Internet concerned vertebrates, in particular medium-sized mammals, but also included a relatively large number of carnivores. This can be explained by the following facts. Firstly, most cases concerned synanthropic (e.g. racoons, hedgehogs, skunks, etc.) and domestic species (dogs, cats, including those that range free) and occurred in urban areas. This may be a result of the common observations of nature in the surroundings of human settlements, increased amount of litter in such areas, as well as an increased number of animal populations that penetrate the anthropogenic environment^51^. One of the reasons is that these animals use waste as food sources^52^. Moreover, it is more likely that animals which are stuck in containers in urban areas are noticed by passersby. However, almost one third of incidents occurred in semi-urban or even natural habitats such as forests (far from cities), deserts or national parks, which indicates that littering is common wherever humans set foot^53–55^. Secondly, they include many charismatic species such as monitor lizards *Varanus* spp., bears *Ursus* spp., Iranian wolf *Canis lupus pallipes* or Indian leopard *Panthera pardus fusca*, which arouse sympathy and compassion in people. They are well known among citizens and often serve as a symbol of biodiversity conservation^56^. On the other hand, a significant percentage of recorded cases come from urban areas, which may prove that growing anthropopression forces them to coexist with humans^57,58^. Such biases as non-random sampling in online media based studies are expected^33,38^, therefore results should be carefully interpreted. We, thus, cannot simply conclude that medium-sized mammals are the most commonly prone to getting stuck in discarded containers. Instead, our results provided a complementary data and allowed us to extended the list of threatened animals beyond small sized animals reported in scientific literature.

Although most trapped animals are within the category of least concern, some are rare at local scale (e.g. red squirrel *Sciurus vulgaris* in Great Britain, the endemic beetle *Entomochilus horatii* and many-spotted tree iguana *Liolaemus nigromaculatus* in Chile). Others are listed as endangered (red-crowned crane *Grus japonensis*, eastern quoll *Dasyurus viverrinus*) and critically endangered (Bermuda rock skink *Plestiodon longirostris*) according to IUCN^59^. One of the main threats to the population of the latter is indeed discarded containers^22^. Despite lower species richness, similar results are also found in the literature^26^; in their study on the mortality of small mammals in bottles and drink cans in the Cherokee National Forest in the United States, 18% of individuals were noted as rare species.

Usually single individuals get trapped in containers, thus this phenomenon should probably not have a significant impact on the population size of a particular species. However, the exceptions are sporadic situations in which a single container can intensively affect local groups of animals, resulting in the death of even several dozen mammals^60^, or when littering occurs on a large scale in a relatively small area, e.g. on islands^22,30^.

So far, bottles^25,31^, drink cans^26^, and cups^61^ have been considered as the most dangerous to animals. However, most records found in our study concerned jars and steel cans. Additionally, in some cases containers originally did not contain foodstuffs but detergents or fuel. Although we cannot exclude the possibility of food remains or small animal carcasses that lured other individuals in, we suggest that animals do not always explore garbage in search of food but also out of curiosity, while playing or while looking for a shelter^27^. Mammals were most common in almost all types of analyzed containers; however, depending on animal size, they were susceptible to different kinds of container. The exception were drink cans in which reptiles dominated; reptiles were also the second most numerous group of animals found in our study. This may be a result of a relatively large number of records from Asia and Australia, where these animals are very common, often live in urban areas^62^, and presumably entered these containers because they offered shelter. It can be concluded that no single type of container can be considered the most dangerous, because different types affect different groups of animals in varying ways. For example, containers with narrow openings such as bottles and drink cans are the most dangerous to invertebrates, small mammals, and reptiles, while containers with wide openings are a danger to medium sized and large mammals. It should be also emphasized that other types of litter not analyzed in this study, e.g. plastic bags, are a lethal threat to fauna. As well as trapping animals, litter poses a threat of injury e.g. fragments of broken glass, ingestion or entanglement^19,63–66^.

In most incidents, citizens tried to help trapped animals, even if it posed a threat to their own health (e.g. the rescue of venomous snakes or dangerous predators). This corresponds with the high level of involvement of volunteers in wildlife rescue^67^ and confirms that they have empathy toward animals^68^. Sometimes the materials shared in social media were noticed by journalists from information portals and, as a result, they reached wider audience, helping to raise public awareness of how discarded debris in the environment can affect animals. Our study has shown that the number of incidents reporting animals getting stuck in containers that were shared on social media has significantly increased in recent years (Fig. 1). The main causes of this phenomenon may be general increase in littering in the environment, human empathy towards suffered animal^69^, as well as an increase in the number of social media users as a result of global accessibility and growing popularity^3,36^. Importantly, social media plays an indispensable role in supplying scientific data related to the protection of nature and the impact of people on the environment^33,34^. For instance, social media has been used to assess the influence of human-impacted habitats on collisions between primates and vehicles^48^, for the monitoring of cetaceans in the central Mediterranean^49^, for mapping the distribution of pollinators and flowering plants in Australia^70^, and as a method of tracking global illegal wildlife trade^71,72^.

## Conclusions

Urbanization, which constitutes a drastic change in land use, has forced animals to live in increasingly littered habitats. Despite its global scope, the problem of the effect of garbage on terrestrial animals is often underestimated or ignored. However, our study shows that discarded containers are deadly traps for animals from small invertebrates to large carnivores and in various habitats including undisturbed wilderness. Assessment of the actual impact of littering on the decline of any particular species, especially globally or regionally threatened, requires further research; data obtained from animal rescue organizations that often help trapped animals^73^ can be helpful.

The scale of littering of terrestrial ecosystems requires immediate action. Policymakers, consumers and industry together must take steps to reduce litter pollution by responsible product management and limitation of consumerism. Recent studies illustrate a significant positive effects of ‘container deposit legislation’ on reducing discarded beverage containers^74^, but still many countries have been not implemented any economic incentives to control land-based litter. Additionally, local scale extensive volunteer cleanup action can contribute to decline in litter in the wider environment (especially beverage containers)^75^. Such actions should be regularly repeated at the same sites, supported (e.g. providing gloves and bin bags, payment of litter disposal) and ideally managed by local government or other institutions^75^. Importantly, participation in such actions also has educational value by strengthening good, pro-environmental habits and raising public awareness^76,77^. Finally, we hope that photos and videos shared through online media raise citizen awareness of the consequences of littering on wildlife.

## Material and methods

### Data collection

Our study material consists of cases documented on online media of animals interacting with discarded containers and encountering difficulties freeing themselves. We manually explored internet platforms such as Google Images, YouTube, Facebook, Instagram and Twitter from July to the end of November 2019. Different combinations of the following key words were used: *animal* (replaced by common name of various taxa) + *dead*/*stuck*/*trapped* + *bottle*/*can*/*container*/*jar*/*tin* (see Supplementary Table S5 online for details). In addition to English, using Google Translate, the search was conducted in seven of the world's most-used languages (Mandarin Chinese, Hindi, Spanish, French, Arabic, Bengali, and Russian; based on Ethnologue^78^) and Polish. Only good quality videos or pictures and only cases in which the animals independently interacted with containers were used. Cases when one author published a photo series documenting a single event, or when the same event was published across different platforms were treated as one record.

Animals were determined to species or the lowest possible taxonomic rank and, if possible, classified according to the International Union for Conservation of Nature Red List category^59^. Mammals were additionally grouped into three categories of size criterion following Lessa and Farina^79^: (i) small—less than 1 kg mass of adult, (ii) medium—between 1 and 100 kg, (iii) large—more than 100 kg. If possible, the number of animals stuck in a single container (in case of most invertebrates, only the estimated number), the locality (continent, country) and habitat type (urban—cities, villages, parks, etc. or natural/seminatural—forests, meadows, deserts, protected areas, etc.; recorded based on photograph/movie or information in the text) were noted.

Containers that were traps for animals were classified as follows: (i) bottle—plastic or glass narrow-necked container for liquid storage, (ii) jar—plastic or glass with a wide opening container used for food storage and preservation, (iii) drink can—single-use aluminum container used for storing beverages or other liquids, (iv) steel can—steel container with a wide opening used for the distribution or storage of goods, e.g. cat/dog food, (v) cup—open-topped plastic or paper container used to hold liquids (including those with lids), (vi) others—containers that do not fit into any of the previous categories (made of glass, metal, plastic, etc. and used for different purposes such as milk or coffee storage). Cases of getting stuck in other litter products (e.g. plastic bags, chips bags, toys, buckets, watering cans, etc.) were excluded from the analysis.

Three categories were used to distinguish the way of being stuck in containers: (i) ‘whole body’—when a whole animal was trapped in a container, (ii) ‘head’—when only the head or head with neck and torso was stuck, (iii) ‘part of body’—when different body parts such as a limb or beak were stuck.

The data collected also took into account whether the animal was alive or dead. If alive, the animal’s condition (with or without injures) was noted, as well as whether it was able to get out of the container on its own, and if not, whether people helped it. This information was recorded only if it was available in the movie or in the photo description.

### Statistical analysis

Spearman's rank correlation coefficient was used to check whether there is a correlation between the publication year and the number of documented reports of animals that got stuck in containers. The chi-square test was used to compare (i) the proportion of containers with trapped animals found in urbanized habitats with those from natural/semi-natural habitats, (ii) the proportion of species classified as of least concern with those classified as endangered (including vulnerable, endangered, critical endangered), (iii) the proportions of particular groups of animals against the type of containers in which they get stuck. The latter analysis was also performed only for mammals. The analyses were performed using Statistica 13.5 (Dell Software). Additionally, we used correspondence analysis (CA) to present graphically the relationships between the most common groups of animals (invertebrates, reptiles, mammals) and different types of discarded containers. This analysis was carried out in R 3.6.2 using factoextra and FactoMineR packages^80,81^.

## Supplementary Information

Supplementary Table S1.

Supplementary Table S2.

Supplementary Table S3.

Supplementary Table S4.

Supplementary Table S5.

## Data Availability

All data analysed during this study are included in this published article (and its Supplementary Information files).

## References

[1] Ravenelle J, Nyhus PJ (2017). Global patterns and trends in human–wildlife conflict compensation. Conserv. Biol..

[2] Hopewell J, Dvorak R, Kosior E (2009). Plastics recycling: Challenges and opportunities. Philos. Trans. R. Soc. B.

[3] Kaza, S., Yao, L., Bhada-Tata, P. & Van Woerden, F. What a waste 2.0: A global snapshot of solid waste management to 2050. *Urban Development* (World Bank, Washington, DC, 2018).

[4] Obradović M, Kalambura S, Smolec D, Jovičić N (2014). Dumping and illegal transport of hazardous waste, danger of modern society. Coll. Antropol..

[5] Kubásek M, Hřebíček J (2013). Crowdsource approach for mapping of illegal dumps in the Czech Republic. Int. J. Spatial Data Infrastruct. Res..

[6] Danthurebandara, M., Van Passel, S., Nelen, D., Tielemans, Y., & Van Acker, K. Environmental and socio-economic impacts of landfills. *Linnaeus Eco-Tech ***2012**, 26–28 (2012).

[7] Lebreton L (2018). Evidence that the Great Pacific Garbage Patch is rapidly accumulating plastic. Sci. Rep..

[8] Baranová B, Manko P, Jászay T (2015). Waste dumps as local biodiversity hotspots for soil macrofauna and ground beetles (Coleoptera: Carabidae) in the agricultural landscape. Ecol. Eng..

[9] Jagiello Z, Dylewski Ł, Tobolka M, Aguirre JI (2019). Life in a polluted world: A global review of anthropogenic materials in bird nests. Environ. Pollut..

[10] Michlewicz M, Tryjanowski P (2017). Anthropogenic waste products as preferred nest sites for *Myrmica rubra* (L.) (Hymenoptera, Formicidae). J. Hymenopt. Res..

[11] Kolenda K (2020). Deadly trap or sweet home? The case of discarded containers as novelty microhabitats for ants. Glob. Ecol. Conserv..

[12] Robertson BA, Rehage JS, Sih A (2013). Ecological novelty and the emergence of evolutionary traps. Trends Ecol. Evol..

[13] Schuyler Q, Hardesty BD, Wilcox C, Townsend K (2014). Global analysis of anthropogenic debris ingestion by sea turtles. Conserv. Biol..

[14] Roman L, Schuyler QA, Hardesty BD, Townsend KA (2016). Anthropogenic debris ingestion by avifauna in eastern Australia. PLoS One.

[15] Zhao S, Zhu L, Li D (2016). Microscopic anthropogenic litter in terrestrial birds from Shanghai, China: Not only plastics but also natural fibers. Sci. Total Environ..

[16] Lusher AL (2015). Microplastic and macroplastic ingestion by a deep diving, oceanic cetacean: The True's beaked whale *Mesoplodon mirus*. Environ. Pollut..

[17] Foley CJ, Feiner ZS, Malinich TD, Höök TO (2018). A meta-analysis of the effects of exposure to microplastics on fish and aquatic invertebrates. Sci. Total Environ..

[18] Rideout BA (2012). Patterns of mortality in free-ranging California Condors (*Gymnogyps californianus*). J. Wildl. Dis..

[19] Strine CT (2014). Mortality of a wild king cobra, *Ophiophagus hannah* Cantor, 1836 (Serpentes: Elapidae) from Northeast Thailand after ingesting a plastic bag. Asian Herpetol. Res..

[20] Ryan PG, Dilley BJ, Ronconi RA, Connan M (2019). Rapid increase in Asian bottles in the South Atlantic Ocean indicates major debris inputs from ships. Proc. Natl. Acad. Sci. USA..

[21] Debernardi P, Patriarca E, Perrone A, Cantini M, Chiarenzi B (1997). Small mammals found in discarded bottles in alpine and pre-alpine areas of NW-Italy. Hystrix.

[22] Davenport J, Hills J, Glasspool A, Ward J (2001). Threats to the critically endangered endemic Bermudian skink *Eumeces longirostris*. Oryx.

[23] Benedict RA, Billeter MC (2004). Discarded bottles as a cause of mortality in small vertebrates. Southeast. Nat..

[24] Brannon MP, Bargelt LB (2013). Discarded bottles as a mortality threat to shrews and other small mammals in the Southern Appalachian Mountains. J. N. C. Acad. Sci..

[25] Morris PA, Harper JF (1965). The occurrence of small mammals in discarded bottles. Proc. Zool. Soc. Lond..

[26] Hamed MK, Laughlin TF (2015). Small-mammal mortality caused by discarded bottles and cans along a US Forest Service road in the Cherokee National Forest. Southeast. Nat..

[27] Kolenda K, Przybył M, Piłacińska B, Rychlik L (2018). Survey of discarded bottles as an effective method in detection of small mammal diversity. Pol. J. Ecol..

[28] Kolenda K, Kurczaba K, Kulesza M (2015). Littering as a lethal threat to small animals. Przegląd Przyr..

[29] Poeta G, Romiti F, Battisti C (2015). Discarded bottles in sandy coastal dunes as threat for macro-invertebrate populations: First evidence of a trap effect. Vie Milieu.

[30] Lavers JL, Sharp PB, Stuckenbrock S, Bond AL (2020). Entrapment in plastic debris endangers hermit crabs. J. Hazard. Mater..

[31] Moates G (2018). Small mammal mortality in discarded bottles and drinks cans—A Norfolk-based field study in a global context. J. Litter Environ. Qual..

[32] Castilla AM, Bauwens D (1991). Observations on the natural history, present status, and conservation of the insular lizard *Podarcis hispanica atrata* on the Columbretes archipelago, Spain. Biol. Conserv..

[33] Di Minin E, Tenkanen H, Toivonen T (2015). Prospects and challenges for social media data in conservation science. Front. Environ. Sci..

[34] Toivonen T (2019). Social media data for conservation science: A methodological overview. Biol. Conserv..

[35] Kaplan AM, Haenlein M (2010). Users of the world, unite! The challenges and opportunities of Social Media. Bus. Horiz..

[36] Perrin A (2015). Social media usage 2005–2015.

[37] Jagiello Z, Dyderski MK, Dylewski Ł (2019). What can we learn about the behaviour of red and grey squirrels from YouTube?. Ecol. Inform..

[38] Ruths D, Pfeffer J (2014). Social media for large studies of behavior. Science.

[39] Anderson AA, Huntington HE (2017). Social media, science, and attack discourse: How Twitter discussions of climate change use sarcasm and incivility. Sci. Commun..

[40] Sorokowski P, Kowal M, Zdybek P, Oleszkiewicz A (2020). Are online haters psychopaths? Psychological predictors of online hating behavior. Front. Psychol..

[41] Mikula P, Hadrava J, Albrecht T, Tryjanowski P (2018). Large-scale assessment of commensalistic–mutualistic associations between African birds and herbivorous mammals using internet photos. PeerJ.

[42] Daume S, Albert M, Von Gadow K (2014). Forest monitoring and social media—Complementary data sources for ecosystem surveillance?. For. Ecol. Manag..

[43] Stafford R (2010). Eu-social science: The role of internet social networks in the collection of bee biodiversity data. PLoS One.

[44] van Zanten BT (2016). Continental-scale quantification of landscape values using social media data. Proc. Natl. Acad. Sci..

[45] Hausmann A (2018). Social media data can be used to understand tourists' preferences for nature-based experiences in protected areas. Conserv. Lett..

[46] Tryjanowski P (2020). Birds drinking alcohol: Species and relationship with people. A review of information from scientific literature and social media. Animals.

[47] Hausmann A (2019). Assessing global popularity and threats to important bird and biodiversity areas using social media data. Sci. Total Environ..

[48] Hetman M, Kubicka AM, Sparks TH, Tryjanowski P (2019). Road kills of non-human primates: A global view using a different type of data. Mammal Rev..

[49] Pace DS (2019). An integrated approach for cetacean knowledge and conservation in the central Mediterranean Sea using research and social media data sources. Aquat. Conserv..

[50] Guinard É, Julliard R, Barbraud C (2012). Motorways and bird traffic casualties: Carcasses surveys and scavenging bias. Biol. Conserv..

[51] Luniak, M. Synurbization–adaptation of animal wildlife to urban development. In *Proceedings of the 4th International Symposium on Urban Wildlife Conservation, Tucson, AZ* (eds. Shaw, W. W. *et al.*) 50–55 (2004).

[52] Soulsbury CD, White PC (2016). Human–wildlife interactions in urban areas: A review of conflicts, benefits and opportunities. Wildlife Res..

[53] Brown TJ, Ham SH, Hughes M (2010). Picking up litter: An application of theory-based communication to influence tourist behaviour in protected areas. J. Sustain. Tour..

[54] Wilson SP, Verlis KM (2017). The ugly face of tourism: Marine debris pollution linked to visitation in the southern Great Barrier Reef, Australia. Mar. Pollut. Bull..

[55] Jakiel M, Bernatek-Jakiel A, Gajda A, Filiks M, Pufelska M (2019). Spatial and temporal distribution of illegal dumping sites in the nature protected area: The Ojców National Park, Poland. J. Environ. Plan. Manag..

[56] Ducarme F, Luque GM, Courchamp F (2013). What are “charismatic species” for conservation biologists. BioSci. Master Rev..

[57] Elfström M, Zedrosser A, Støen OG, Swenson JE (2014). Ultimate and proximate mechanisms underlying the occurrence of bears close to human settlements: Review and management implications. Mammal Rev..

[58] Kumbhojkar S, Yosef R, Benedetti Y, Morelli F (2019). Human-leopard (*Panthera pardus fusca*) co-existence in Jhalana forest reserve, India. Sustainability.

[59] IUCN. The IUCN Red List of Threatened Species, http://www.iucnredlist.org (2019).

[60] Arrizabalaga A, González LM, Torre I (2016). Small mammals in discarded bottles: A new world record. Galemys.

[61] Chandrasekaran S (2011). Disposed paper cups and declining bees. Curr. Sci..

[62] Shine R, Koenig J (2001). Snakes in the garden: An analysis of reptiles “rescued” by community-based wildlife carers. Biol. Conserv..

[63] Peris SJ (2003). Feeding in urban refuse dumps: Ingestion of plastic objects by the White Stork (*Ciconia ciconia*). Ardeola.

[64] Mrosovsky N, Ryan GD, James MC (2009). Leatherback turtles: The menace of plastic. Mar. Pollut. Bull..

[65] Jankowiak Ł, Malecha AW, Krawczyk AJ (2016). Garbage in the diet of carnivores in an agricultural area. Eur. J. Ecol..

[66] Poeta G, Eleonora S, Alicia TR, Battisti C (2017). Ecological effects of anthropogenic litter on marine mammals: A global review with a “black-list” of impacted taxa. Hystrix.

[67] Heathcote G, Hobday AJ, Spaulding M, Gard M, Irons G (2019). Citizen reporting of wildlife interactions can improve impact-reduction programs and support wildlife carers. Wildlife Res..

[68] Fraser H, Taylor N, Signal T (2017). Young people empathising with other animals: Reflections on an Australian RSPCA humane education programme. Aotearoa N. Z. Soc. Work.

[69] Tiplady CM, Walsh DAB, Phillips CJ (2013). Public response to media coverage of animal cruelty. J. Agric. Environ. Ethics..

[70] ElQadi M (2017). Mapping species distributions with social media geo-tagged images: Case studies of bees and flowering plants in Australia. Ecol. Inform..

[71] Siriwat P, Nijman V (2018). Illegal pet trade on social media as an emerging impediment to the conservation of Asian otters species. J. Asia-Pacific Biodivers..

[72] Di Minin E, Fink C, Hiippala T, Tenkanen H (2019). A framework for investigating illegal wildlife trade on social media with machine learning. Conserv. Biol..

[73] RSPCA. Plastic litter is a growing threat to animals reveals RSPCA Cymru, https://news.rspca.org.uk/2019/02/05/plastic-litter-is-a-growing-threat-to-animals-reveals-rspca-cymru/ (2019).

[74] Schuyler Q, Hardesty BD, Lawson TJ, Opie K, Wilcox C (2018). Economic incentives reduce plastic inputs to the ocean. Mar. Policy.

[75] Haarr ML, Pantalos M, Hartviksen MK, Gressetvold M (2020). Citizen science data indicate a reduction in beach litter in the Lofoten archipelago in the Norwegian Sea. Mar. Pollut. Bull..

[76] Brannon MP, Brannon JK, Baird RE (2017). Educational applications of small-mammal skeletal remains found in discarded bottles. Southeast. Nat..

[77] Wyles KJ, Pahl S, Holland M, Thompson RC (2017). Can beach cleans do more than clean-up litter? Comparing beach cleans to other coastal activities. Environ. Behav..

[78] Ethnologue 2019. What are the top 200 most spoken languages? http://www.ethnologue.com/guides/ethnologue200 (2019).

[79] Lessa EP, Farina RA (1996). Reassessment of extinction patterns among the late Pleistocene mammals of South America. Palaeontology.

[80] Lê S, Josse J, Husson F (2008). FactoMineR: An R package for multivariate analysis. J. Stat. Softw..

[81] Kassambara, A. & Mundt, F. factoextra: Extract and Visualize the Results of Multivariate Data Analyses. R package version 1.0.7. https://CRAN.R-project.org/package=factoextra (2020).

