# Supplementary material for: Online media reveals a global problem of discarded containers as deadly traps for animals
Source: Sci Rep. 2021 Jan 11;11:267. doi: 10.1038/s41598-020-79549-8 (PMC7801720; doi:10.1038/s41598-020-79549-8)
Supplement: Supplementary file 1 — Supplementary Table S1. [file 41598_2020_79549_MOESM1_ESM.pdf]

Online media reveals a global problem of discarded containers as deadly traps for animals
Krzysztof Kolenda, Monika Pawlik, Natalia Kuśmierek, Adrian Smolis, Marcin Kadej

Supplementary Table1. Links to reports on the animals getting stuck in discarded containers shared on social media.

Link	Access date	Trapped animal
http://www.deadlinenews.co.uk/2018/07/26/endangered-red-squirrel-found-dead-trapped-inside-abandoned-plastic-jar/	01.07.2019	*Sciurus vulgaris*
https://www.facebook.com/torontowildlifecentre/posts/10156320837988656	01.07.2019	*Procyon lotor*
https://patch.com/massachusetts/chelmsford/skunked-beer-animal-rescued-after-getting-head-stuck-can	01.07.2019	*Mephitis mephitis*
https://imgur.com/gallery/gCI3W	02.07.2019	*Masticophis lateralis*
https://www.arlboston.org/low-stress-approach-removing-jar-raccoons-head/	02.07.2019	*Procyon lotor*
https://www.thedodo.com/in-the-wild/possum-trapped-nutella-jar-australia?fbclid=IwAR2CnxOjZiNFWCstTzH2laAdZtDqNkNuNkRmJ5k86ccLevhQEYValRdvl_Q	02.07.2019	*Anas platyrhynchos*
https://www.itv.com/news/central/2015-05-18/duck-suffers-horrific-injuries-as-beak-gets-stuck-in-can/	03.07.2019	*Trichosurus vulpecula*
https://timesofindia.indiatimes.com/city/bhubaneswar/frantic-search-to-locate-stray-with-plastic-jar-stuck-on-head/articleshow/61790784.cms	04.07.2019	*Naja sumatrana*
https://www.youtube.com/watch?v=fKhpNZoc0OE	05.07.2019	*Erinaceus europaeus*
https://stock.adobe.com/ee/editorial/a-dog-with-a-plastic-container-stuck-in-its-head-walks-in-the-rebel-held-besieged-town-of-douma-eastern-damascus-suburb-of-ghouta/156894520	04.07.2019	*Paguroidea*
https://m.facebook.com/story.php?story_fbid=1238064216332666&id=264705513668546	04.07.2019	*Ursus americanus*
https://l.facebook.com/l.php?u=https%3A%2F%2Fwww.bbc.co.uk%2Fnewsround%2F18205764%3Ffbclid%3DIwAR3hCiGqAhCqNZOJ42GX1BpJpV0q93fTyVA4_j-ViSVvjyALYzeJTjc_MWQ&h=AT0LIsvndD1d0hqerq4idKQeIGJ-tN8UVC_Y1YeMYPAe9ATJpyVjVBe7HeXokh2w93	04.07.2019	*Erinaceus europaeus*

6Ynu2b9F6JF5XNdqdbXF4n14N6Bvrl-UbaOpqLqSOliYxW_e0Fr6DVPw3duG3Ou9PU		
https://www.youtube.com/watch?v=CQYoD3_leKU&feature=share&fbclid=IwAR3rcaKuiBujF2cVoa3QVL1dT3FJbzafTImHaQO4DugupEnDYAdqMImPA9E	04.07.2019	*Panthera pardus fusca*
https://m.facebook.com/story.php?story_fbid=968733509950035&id=138964782926916	05.07.2019	*Canis latrans*
https://m.facebook.com/browardcturtles/posts/1830984393647842?refsrc=http%3A%2F%2Fcbs12.com%2Fnews%2Flocal%2Fphoto-loggerhead-sea-turtle-hatchling-stuck-in-cup	05.07.2019	*Caretta caretta*
https://www.valleycenter.com/articles/coyote-killed-by-discarded-bottle-inspires-residents-to-hold-cleanup/?fbclid=IwAR0BVPLhO91B2cOq5tG0oh-eL51jpLobC6gF-7_rQdossMYsjPxt4aWDKL0	05.07.2019	*Canis latrans*
https://l.facebook.com/l.php?u=https%3A%2F%2Fwww.alaskapublic.org%2F2016%2F06%2F14%2Fbear-gets-head-stuck-in-can-officials-jump-to-aid%2F%3Ffbclid%3DIwAR01OAHNbO0GOTMvdpJjPZmb89JJxFGLyntrbeBtgSMI5pD5zbhqWzkjZpk&h=AT0c-b7yTq61ZMaFw3d6QOKcrZfTUvfzpYbRloOmYHTESqkQmc1E1zaP5bli1AC55tIGGw1x9A0zd8oDBPIV3G5pUAuXWHzqX0nonFgbxWy0g18CnPW_oMbXP-mhAvoa5mQAjw	05.07.2019	*Ursus americanus*
https://oasisresortbelize.wordpress.com/?s=Frog&submit=Search&fbclid=IwAR21qmYh3g1kv80LfPlmIpzRYjKlFkzdV64WCqfsbPAjwYvyMXVr0q0s1qg	05.07.2019	*Rhinella marina*
https://amp.news.com.au/national/south-australia/lizard-stuck-in-beer-can-prompts-wildlife-litter-warning/news-story/c6abf0bc4ef0741163817354d1f2ef48?fbclid=IwAR0FWMSNuy213LT_v1XY6WvIqkmbm2jwcS_XxCiADzytN8BkXyQw74nyfI	05.07.2019	*Varanus tristis*
http://www.reptilesmagazine.com/Police-in-Australia-Goes-Beyond-Call-of-Duty-to-Save-Lizard-Stuck-in-Red-Bull-Can/?fbclid=IwAR3vSO2o3T2sMYuWlbPAMNSYy5tvs0MksnrzFbUjKqWbrqLOGIIkUaeJ7gY	05.07.2019	*Varanus gouldii*
https://www.thecourier.com.au/story/3478898/snake-gets-stuck-in-can-after-bourbon bender/?fbclid=IwAR31ccK4LbajssbsENz7e4DaK73SqvXXXQ1FheC3Y8TwzKYkfIKmqeD4NKQ	05.07.2019	*Austrelaps* sp.
https://metro.co.uk/2018/07/05/badly-malnourished-wolf-close-death-plastic-container-gets-stuck-head 7687348/?fbclid=IwAR1asCW_qK1VF2XM303M75wNoe4U_Xhp8cZjZIcfs9LKsYhrjKGEx8jWpo https:	05.07.2019	*Canis lupus pallipes*

//www.unilad.co.uk/life/starving-wolf-rescued-after-getting-head-stuck-in-container/		
https://www.youtube.com/watch?v=IFVg4PVJ6Y&feature=share&fbclid=IwAR0K_ztq2jjd7dhdTw8vTGByN_fkosCMGaQMnwMNnKgcdJ4WH4P3RKHQjcI	05.07.2019	*Vulpes vulpes*
https://www.petethomasoutdoors.com/2013/06/deer-with-plastic-jar-stuck-on-its-head-in-m/innesota-gets-helping-hand.html?fbclid=IwAR1IKeMFTQpKaju_NrGhFTODuSPxk4hmnanH-Uz812A4-e42ITffB4kza1Q	08.07.2019	*Odocoileus virginianus*
https://www.youtube.com/watch?v=fK5IpP50oZM&feature=share&fbclid=IwAR2WRwOYi8qLjO-MUavbNwcdfViJ-nS4tJqbrOskmwSe-xZKlmQWdYMLWHk	08.07.2019	*Notechis scutatus*
https://fox5sandiego.com/2016/01/11/coyote-with-jar-stuck-on-head-found-in-bonita/?fbclid=IwAR3IoggkvmdAhxST19g4nS3BuaYfj8lzBRO1PZHW6lOTBfzbF-Q1HRW-utU	09.07.2019	*Canis latrans*
https://m.facebook.com/photo.php?fbid=1867070853309065&id=100000185049076&set=a.314561151893384&source=48	10.07.2019	*Sciurus carolinensis*
https://www.dailymail.co.uk/news/article-6105611/Russian-man-risks-life-trying-free-bear-head-stuck-giant-tin-Yakutia-region.html?fbclid=IwAR39_VGiEQrTkCVQK7ASSmZdU9umKUQnI6q0sm05P8Sm1TQQjvBhbH_fpiE	10.07.2019	*Ursus arctos*
https://www.tvnz.co.nz/one-news/new-zealand/-another-life-saved-police-come-to-rescue-of-hedgehog-with-mcflurry-container-stuck-on-its-head-6133644	10.07.2019	*Erinaceus europaeus*
https://www.facebook.com/newlondon.animalcontrol/photos/a.1588816804708174/1887471474842704/?type=3	11.07.2019	*Procyon lotor*
https://www.treehugger.com/natural-sciences/fox-cub-head-stuck-jar-approaches-humans-help.html	11.07.2019	*Vulpes vulpes*
http://www.royalgazette.com/article/20150702/NEWS07/150709939	11.07.2019	*Plestiodon longirostris*
http://www.saveourwaterwaysnow.com.au/01_cms/details.asp?ID=538	13.07.2019	*Cyclodomorphus gerrardii*
https://twitter.com/karenin55/status/1123342012273631233	13.07.2019	*Lacerta agilis*
https://www.arc-trust.org/news/litter-in-the-countryside-its-rubbish-for-amphibians-and-reptiles-too	10.09.2019	*Zootoca vivipara*
https://petinformers.com/2018/12/15/raccoon-looking-for-a-snack-gets-his-head-stuck-in-a-can/	13.07.2019	*Procyon lotor*
https://www.dailymail.co.uk/news/article-	13.07.2019	*Pseudechis*

2830876/Snake-Red-bellied-black-snake-saved-truckie-cut-free-head-got-stuck-energy-drink-can.html		*porphyriacus*
https://www.dailymail.co.uk/news/article-5639997/Divers-rescue-turtle-shell-deformed-plastic-container.html	13.07.2019	*Cheloniidae*
https://www.youtube.com/watch?v=lJU-ag3EZsw	13.07.2019	*Ursus arctos*
https://www.youtube.com/watch?v=NC55Kix3aCU	13.07.2019	*Macaca* sp.
https://www.youtube.com/watch?v=kQAK-8spddw	17.07.2019	*Ursus arctos*
https://www.youtube.com/watch?v=xZuBt9ogwX4	18.07.2019	*Procyon lotor*
https://www.youtube.com/watch?v=Wu6nKXcsf7g	18.07.2019	*Ursus americanus*
https://www.youtube.com/watch?v=vUoluxb5C3U	18.07.2019	*Procyon lotor*
https://www.youtube.com/watch?v=JPiw-cjCbXQ	18.07.2019	*Procyon lotor*
https://www.youtube.com/watch?v=yLz4MYNLnxI	18.07.2019	*Mephitis mephitis*
https://www.youtube.com/watch?v=sjeSacbW0Eg	18.07.2019	*Odocoileus* sp.
https://www.youtube.com/watch?v=DQMFwJqPYXI	18.07.2019	*Procyon lotor*
https://www.youtube.com/watch?v=6P9Uz8N_sP8	18.07.2019	*Ursus americanus*
https://www.dailymail.co.uk/news/article-3101198/Fantastic-Mr-Fox-Heartwarming-moment-seven-week-old-cub-head-stuck-plastic-bottle-gets-cut-free-animal-rescue-team.html	18.07.2019	*Vulpes vulpes*
https://www.huffpost.com/entry/black-bear-saved-plasticcontainer_n_5791c5b3e4b0bdddc4d4001d?guccounter=1&guce_referrer=aHR0cDovL3d3dy5ib3NoZW1pYXNib2hlbWlhLmNvbS9tYW4tbGFzc29lcy1iZWFyLXdpdGgtanVnLW9uLWl0cy1oZWFkLWJlY2F1c2UtY29sb3JhZG8v&guce_referrer_sig=AQAAAFewliX20vT4N0wGcD_3P_T1_xEIG_Yd3rvEvykJ4e4ty7N9fvhf2RyA-3ZlE1kyM8rVP3KBLew-ulvwN3C7kwPsyWFbvznC6mBhcvbDzZXMNl0bd0-MTwLGymCwtjCWHD-FW5NMpO4xg-EDY2YPK3EtBo2_ax5pmkJ3oxDHZ8Vk	18.07.2019	*Ursus americanus*
https://www.youtube.com/watch?v=lexmOq-ZMTY	19.07.2019	*Procyon lotor*
https://www.cbs58.com/news/coyote-saved-after-plastic-container-was-stuck-on-his-head-for-a-week	28.07.2019	*Canis latrans*
https://www.abc.net.au/news/2019-01-23/plastic-waste-found-in-quoll-scats-in-tasmania/10731452	28.07.2019	*Dasyurus viverrinus*
https://www.youtube.com/watch?v=hlEpFuVISno&feature=share&fbclid=IwAR0Uuj6FKmV9MNOuQTWmk2NnCE_4axWM7PqFO-HkQNp1wrytdzIkItizcdI	28.07.2019	*Canis lupus familiaris*
https://www.youtube.com/watch?v=8L6nIzuwghs bottle-for-three-weeks/	10.08.2019	*Canis lupus familiaris*
https://twitter.com/hodgeyCafc/status/1075366038441811968	10.08.2019	*Apodemus sylvaticus*
https://www.mapleridgenews.com/news/bear-conflicts-on-the-rise-in-north-fraser/	10.08.2019	*Ursus americanus*
https://www.independent.ie/world-news/and-finally/curious-raccoon-rescued-by-police-after-	10.08.2019	*Procyon lotor*

getting-head-stuck-in-jar-38383341.html		
https://www.theguardian.pe.ca/news/local/veterinarian-removes-squirrel-stuck-in-juice-bottle-101716/	10.08.2019	*Sciurus vulgaris*
https://www.bluemountainsgazette.com.au/story/6212131/possum-needed-a-bit-of-police-magic-for-rescue-from-glass-jar/#slide=2	10.08.2019	*dendroaspis polylepis*
https://news.rspca.org.uk/2018/11/09/hedgehog-rescued-from-prickly-situation-after-getting-head-stuck-in-smashed-glass-bottle/	10.08.2019	*Erinaceus europaeus*
https://www.facebook.com/photo.php?fbid=2058677897551258&set=pcb.2058724980879883&type=3&theater&ifg=1	07.08.2019	*Nyctereutes procyonoides*
https://tvnmeteo.tvn24.pl/informacje-pogoda/swiat,27/niedzwiedz-z-glowa-w-blaszanej-bance-musial-blakac-sie-po-lesie-cztery-piec-dni,289096,1,0.html	07.08.2019	*Ursus arctos*
https://www.youtube.com/watch?v=UCbyJvbYrmY	07.08.2019	*Apodemus flavicollis*
https://www.youtube.com/watch?v=7o5aisetJDY	07.08.2019	*Anoplotrupes stercorosus, Carabus coriaceus*
http://wroclaw.wyborcza.pl/wroclaw/7,35771,24697225,sarna-z-plastikowa-butelka-na-glowie-blaka-sie-po-wroclawskim.html	07.08.2019	*Capreolus capreolus*
https://www.facebook.com/NadlesnictwoTuszyma/photos/pcb.515721352195632/515721028862331/?type=3&theater	07.08.2019	*Meles meles*
https://www.facebook.com/MagazynPrzyrodniczySALAMANDRA/photos/a.221092317904889/2904083666272394/?type=3&theater	07.08.2019	*Vulpes vulpes*
https://www.earth.com/news/emaciated-bear-plastic-container/	27.08.2019	*Ursus americanus*
https://www.agefotostock.com/age/en/Stock-Images/Rights-Managed/FHR-90100-00075-012	27.08.2019	*Mustela nivalus*
https://www.canstockphoto.com/dead-rat-in-a-jar-61610658.html	27.08.2019	*Rattus* sp.
https://www.independent.co.uk/news/uk/home-news/fox-stuck-rspca-rescue-knock-door-plastic-tub-a8855701.html#comments<br>https://www.belfasttelegraph.co.uk/news/viral/fox-found-with-its-head-stuck-inside-peanut-butter-jar-38053719.html	27.08.2019	*Vulpes vulpes*
https://www.deviantart.com/valaquendi/art/Dead-mouse-in-a-bottle-317750642	27.08.2019	*Murinae*
https://www.alamy.com/dead-mouse-trapped-in-beer-bottle-image1754961.html	27.08.2019	*Murinae*
https://drive.google.com/open?id=11cwOqyA0njOPnq	30.08.2019	*Podarcis muralis*

6oep4ZnLvAs29G4S-z		
https://www.reddit.com/r/AnimalsBeingDerps/comments/8m2lm7/ground_lizard_stuck_in_sprite_can_i_got_it_out/	01.09.2019	Squamata
https://vinepair.com/booze-news/snake-beer-cans/	01.09.2019	*Pseudechis porphyriacus*
https://www.youtube.com/watch?v=h5P3OZi5Pgw	01.09.2019	*Pituophis catenifer*
https://www.dailytelegraph.com.au/news/nsw/snake-in-a-beer-can-have-you-ever-seen-anything-more-australian/news-story/98022198e975a813107885dc5f69aa6c	01.09.2019	*Pseudechis porphyriacus*
https://www.youtube.com/watch?v=utfRcxmDMLY	01.09.2019	Serpentes
https://www.youtube.com/watch?v=2dhjqwuJ3dY; https://www.mangalorean.com/for-the-good-times-cheers-snake-got-stuck-inside-a-kf-beer-can/	01.09.2019	*Naja naja*
https://www.alamy.com/stock-photo-red-bellied-black-snake-killed-after-being-stuck-in-a-beer-can-victoria-124553644.html	01.09.2019	*Pseudechis porphyriacus*
http://pressonline.vn/cuu-ran-ket-dau-trong-lon-nuoc-ngot-thay-su-la-p-16687.pol	01.09.2019	*Austrelaps superbus*
https://www.telegraph.co.uk/news/earth/earthpicturegalleries/8915882/Animal-pictures-of-the-week-25-November-2011.html?image=7	01.09.2019	*Varanus tristis*
https://www.wptv.com/news/state/florida-woman-rescues-snake-stuck-in-beer-can-video	01.09.2019	*Coluber constrictor*
https://www.dailymail.co.uk/news/article-2997140/Caught-act-Brown-snake-gets-head-stuck-beer-can.html	01.09.2019	*Pseudonaja textilis*
https://www.thelondoneconomic.com/must-reads/cobra-rescued-spending-four-days-head-stuck-beer-can/16/10/	01.09.2019	*Naja naja*
https://www.google.com/imgres?imgurl=https%3A%2F%2Fcdn2img.pressreader.com%2Fpressdisplay%2Fdocserver%2Fgetimage.aspx%3FregionKey%3D5NAmjuPYnmpYD0jo1UfI4Q%253D%253D&imgrefurl=https%3A%2F%2Fwww.pressreader.com%2Faustralia%2Falbany-advertiser%2F20171205%2F281487866678318&docid=vaIajbnGlISLbM&tbnid=LYsAQPtR3MkLUM%3A&vet=10ahUKEwjKv7rO7q_kAhUa6aYKHfmXAHoQMwh_KEcwRw..i&w=420&h=357&itg=1&client=firefox-b-d&bih=728&biw=1536&ved=0ahUKEwjKv7rO7q_kAhUa6aYKHfmXAHoQMwh_KEcwRw&iact=mrc&uact=8	01.09.2019	*Pseudonaja affinis*
https://www.abc.net.au/news/2012-03-29/mulgasnake.jpg/3919302	01.09.2019	*Pseudechis australis*

https://www.dailymail.co.uk/news/article-5662481/Red-bellied-black-snake-lucky-alive-handler-cuts-head-discarded-soft-drink.html	01.09.2019	*Pseudechis porphyriacus*
https://www.dailytelegraph.com.au/newslocal/city-east/redbellied-black-found-in-a-soft-drink-can-prompts-warning-about-the-start-of-sydneys-snake-season/news-story/d2f0b007aad8e9d89d61f4eecb3d886b	01.09.2019	*Pseudechis porphyriacus*
https://www.abc.net.au/news/2019-01-31/snake-gets-energy drink-can-stuck-on-head/10767774	01.09.2019	*Austrelaps superbus*
https://www.dailymail.co.uk/news/article-5309959/Dead-red bellied-black-snake-stuck-Red-Bull-can.html	01.09.2019	*Pseudechis porphyriacus*
https://www.stuff.co.nz/world/americas/84770779/businessman-rescues-skunk-with-its-head-stuck-in-a-coke-can	01.09.2019	*Mephitis mephitis*
https://globalnews.ca/video/3551987/ont-woman-comes-to-the-aid-of-skunk-with-cup-stuck-on-its-head	01.09.2019	*Mephitis mephitis*
https://www.stuff.co.nz/oddstuff/65672348/	01.09.2019	*Erinaceus europaeus*
https://www.dw.com/en/hedgehog-rescued-in-bavaria-after-getting-stuck-in-plastic-cup/a-43660658	01.09.2019	*Erinaceus europaeus*
http://homepage.eircom.net/~foxwatchireland/rubbish.htm	01.09.2019	*Vulpes vulpes*
https://news.sky.com/story/grumpy-bear-cub-gets-head-stuck-in-cookie-jar-10398381	01.09.2019	*Ursus americanus*
https://www.stuff.co.nz/world/americas/71248753/	01.09.2019	*Ursus americanus*
http://edition.cnn.com/2010/US/08/15/florida.bear.freed/index.html	01.09.2019	*Ursus americanus*
https://www.cbs58.com/news/bucket-head-bear-saved-after-walking-around-with-jug-on-its-head-for-weeks	02.09.2019	*Ursus americanus*
https://ananova.news/fox-cub-with-head-stuck-in-plastic-rubbish-rescued/	02.09.2019	*Vulpes vulpes*
https://www.flickr.com/photos/riverrelief/4624462039	02.09.2019	*Ictalurus furcatus*
https://www.newsbook.com.mt/artikli/2018/07/01/his-head-was-stuck-in-a-plastic-bottle-it-broke-my-heart/?lang=en	02.09.2019	*Erinaceus europaeus*
https://www.youtube.com/watch?v=Tb7pv1iytug	02.09.2019	*Vulpes vulpes*
http://theanimal-zone.blogspot.com/2011/07/whiskers-in-jar-nosey-fox-gets-head.html	02.09.2019	*Vulpes vulpes*
https://mynorthwest.com/1158402/man-regrets-reporting-coyote-with-head-stuck-in-container-after-animal-is-shot-and-killed/	02.09.2019	*Canis latrans*
https://www.telegraph.co.uk/news/worldnews/northamerica/usa/2478421/Bear-with-jar-on-head-shot-dead.html	02.09.2019	*Ursus americanus*
https://weather.com/science/nature/video/wildlife-	02.09.2019	*Ursus americanus*

officials-rescue-bear-cub-with-head-stuck-in-plastic-jar		
https://www.dailyedge.ie/bear-survives-for-three-weeks-with-a-jar-on-its-head-183888-Jul2011/	02.09.2019	*Ursus americanus*
https://m.facebook.com/story.php?story_fbid=1007436726089491&id=339446112888559	09.09.2019	*Apodemus agrarius*
https://www.alamy.com/stock-image-a-lizard-that-has-died-after-becoming-stuck-in-a-plastic-bottle-left-160574128.html	09.09.2019	Squamata
https://www.washingtonpost.com/news/local/wp/2017/01/30/jughead-the-deer-played-hard-to-get-but-its-finally-freed-from-plastic-prison/	09.09.2019	*Odocoileus* sp.
https://www.deeranddeerhunting.com/articles/deer-news/deer-stuck-in-jar	09.09.2019	*Odocoileus* sp.
https://www.krcc.org/post/confronting-urban-deer-population-colorado-springs	09.09.2019	*Odocoileus* sp.
https://www.thedodo.com/in-the-wild/firefighters-help-fox-with-head-in-jar	09.09.2019	*Urocyon cinereoargenteus*
https://www.discoverwildcare.org/skunk-with-a-jar-on-his-head/	09.09.2019	*Mephitis mephitis*
https://www.wideopenspaces.com/wildlife-officials-remove-peanut-butter-jar-from-does-head/	09.09.2019	*Odocoileus* sp.
https://www.youtube.com/watch?v=ajKaTvfD5gE	09.09.2019	*Pytas mucosa*
https://www.couriermail.com.au/news/curious-snake-rescued-after-getting-stuck-in-vb-beer-can-in-blackwood/news-story/df2efcb9159d7861d2e73d9887c3	09.09.2019	*Austrelaps superbus*
https://www.youtube.com/watch?v=updBg8M-0Yg	09.09.2019	Serpentes
https://www.youtube.com/watch?v=4geVWXz05nE	09.09.2019	*Dendroaspis polylepis*
http://www.outdooroddities.com/2012/07/12/snake-with-a-can-on-its-head/	09.09.2019	Serpentes
http://www.birdsinbackyards.net/forum/Rescued-Snake	09.09.2019	*Austrelaps superbus*
http://www.fieldherpforum.com/forum/viewtopic.php?t=19337	09.09.2019	*Pituophis catenifer*
http://landforwildlifealicesprings.blogspot.com/2011/03/spiny-tailed-monitor-varanus-acanthurus.html	09.09.2019	*Varanus acanthurus*
https://www.abc.net.au/news/2017-08-28/snake-with-head-stuck-in-a-can-karratha-2008/8849936	09.09.2019	Serpentes
https://www.youtube.com/watch?v=j7LMJQT8lC4	10.09.2019	*Procyon lotor*
http://natural-history-journal.blogspot.com/2017/07/kick-plastic-habit-plastic-water.html	10.09.2019	*Canis latrans*
https://eu.jsonline.com/story/news/local/lake-country reporter/2017/02/21/plastic-food-containers-pose-threat-animals/98195798/c383	10.09.2019	*Odocoileus* sp.

https://www.seattletimes.com/seattle-news/chase-continues-for-coyote-with-head-stuck-in-jar/	10.09.2019	*Canis latrans*
http://www.adilimad.com/a-dying-mother-goes-as-far-as-she-can-to-save-her-unborn-kittens/?utm_source=&utm_medium=&utm_campaign=&utm_term=&utm_content=	10.09.2019	*Felis catus*
https://www.gon.com/news/coyote-jar-throat	10.09.2019	*Canis latrans*
https://www.peta.org/features/litter-kills-animals/	10.09.2019	*Plestiodon* sp.
https://markgelbart.wordpress.com/2015/11/12/discarded-cans-and-bottles-are-death-traps-for-shrews/	10.09.2019	*Sorex trowbridgii, Diplopoda, Coleoptera*
https://www.mtdemocrat.com/news/yet-another-jar-headed-animal-in-el-dorado-county/attachment/ben-nuckolls_w/	10.09.2019	*Urocyon cinereoargenteus*
https://www.dailypost.co.uk/news/north-wales-news/rspca-warning-cats-heads-stuck-2770619	10.09.2019	*Felis catus*
https://www.mbl.is/ferdalog/frettir/2019/03/04/swan_with_tin_can_stuck_to_beak_being_rescued/	10.09.2019	*Cygnus cygnus*
https://www.bbc.com/news/world-europe-37982402	10.09.2019	*Ursus maritimus*
https://www.nbcboston.com/news/local/Feral-Cat-Raynham-Massachusetts-Tin-Can-Stuck-435855463.html	10.09.2019	*Felis catus*
https://www.euroweeklynews.com/2018/10/09/plastic-trap-distressing-photo-of-deer-goes-viral-in-spain/	10.09.2019	*Capreolus capreolus*
https://www.facebook.com/pumpherstoncc/posts/this-hedgehog-died-stuck-in-a-cup-have-a-wee-think-about-that-next-time-you-drop/2300578149973234/	10.09.2019	*Erinaceus europaeus*
http://www.exnora.org/plastic/animals-sufferings.html	10.09.2019	Phocidae
https://mashable.com/2016/05/20/fox-rescued-peanut-butter-jar/?europe=true	11.09.2019	*Vulpes vulpes*
https://people.com/pets/raccoon-stuck-in-peanut-butter-jar-rescued/	11.09.2019	*Procyon lotor*
http://explorevenango.com/say-what-398/	11.09.2019	*Procyon lotor*
https://www.blogto.com/city/2018/08/toronto-raccoon-peanut-butter-jar/	11.09.2019	*Procyon lotor*
https://buffalonews.com/2014/06/18/raccoon-with-head-stuck-in-peanut-butter-jar-rescued-and-released/	11.09.2019	*Procyon lotor*
https://www.youtube.com/watch?v=xBJAWkLO7FE	11.09.2019	*Procyon lotor*
https://patch.com/connecticut/westport/westport-firefighters-rescue-raccoon-stuck-peanut-butter-jar	11.09.2019	*Procyon lotor*
https://www.arlboston.org/low-stress-approach-removing-jar-raccoons-head/	11.09.2019	*Procyon lotor*
https://www.bbc.com/news/uk-england-manchester-48068138	11.09.2019	*Vulpes vulpes*
http://fourleggedfriendsandenemies.blogspot.com/2016/02/california-petaluma-animal-control.html	11.09.2019	*Mephitis mephitis*
https://twitter.com/iowadnr/status/8877750614821068	11.09.2019	*Procyon lotor*

80		
https://www.canoelover.com/of-raccoons-and-peanut-butter-jars/	11.09.2019	*Procyon lotor*
https://wset.com/news/local/danville-police-officer-helps-rescue-cat-with-head-stuck-in-peanut-butter-jar	11.09.2019	*Felis catus*
https://news.rspca.org.uk/2017/01/25/butter-be-careful-cat-rescued-after-getting-head-stuck-in-jar-of-peanut-butter/	11.09.2019	*Felis catus*
https://www.youtube.com/watch?v=gDB_T1YJYfQ	11.09.2019	*Felis catus*
https://wkow.com/news/2019/06/14/wildlife-rescue-caring-for-bear-after-removing-jar-from-its-head/	11.09.2019	*Ursus americanus*
https://globalnews.ca/news/5055505/skunk-plastic-cup-on-head/	11.09.2019	*Mephitis mephitis*
https://6abc.com/pets-animals/officer-rescues-skunk-with-head-stuck-in-cup/5265941/	11.09.2019	*Mephitis mephitis*
https://www.cbc.ca/news/canada/toronto/skunk-saved-from-toronto-traffic-as-iced-capp-removed-1.1370101	11.09.2019	*Mephitis mephitis*
https://www-1.kansas.com/news/nation-world/national/article234272982.html	11.09.2019	*Mephitis mephitis*
https://www.bostonglobe.com/metro/regionals/south/2017/09/01/when-skunks-crashed-pool-party/dOBvrA3GxEkEjpDPTXpNNO/story.html	11.09.2019	*Mephitis mephitis*
http://arbroath.blogspot.com/2012/09/skunk-gets-head-stuck-in-mayonnaise-jar.html	11.09.2019	*Mephitis mephitis*
https://www.cbc.ca/news/canada/calgary/calgary-skunk-peanut-butter-1.3784147	11.09.2019	*Mephitis mephitis*
https://abc7chicago.com/pets-animals/aurora-police-officer-rescues-skunk-stuck-in-cup/5519443/	11.09.2019	*Mephitis mephitis*
https://www.telegraph.co.uk/news/newstopics/howaboutthat/6325873/Skunk-whisperer-in-peanut-butter-rescue.html	11.09.2019	*Mephitis mephitis*
https://buffalonews.com/2018/09/06/young-skunk-chasing-ice-cream-treat-is-rescued-by-dec/	11.09.2019	*Mephitis mephitis*
https://time.com/3393974/skunk-head-stuck-in-geer-can-fraternity-miami-university-ohio/	11.09.2019	*Mephitis mephitis*
https://www.cbc.ca/news/canada/british-columbia/skunk-with-head-stuck-in-jar-rescued-by-b-c-woman-1.2736261	11.09.2019	*Mephitis mephitis*
https://www.thestar.com/news/canada/2018/10/04/new-brunswick-man-rescues-skunk-on-video-it-could-have-ended-a-lot-worse.html	11.09.2019	*Mephitis mephitis*
https://www.nhregister.com/news/article/Shelton-skunk-tale-shows-negative-effects-of-11560621.php	11.09.2019	*Mephitis mephitis*
https://www.dailymail.co.uk/news/article-6183919/A-squirrel-rescued-trapped-head-stuck-inside-yoghurt-pot.html	11.09.2019	*Sciurus carolinensis*
https://www.nj.com/hunterdon/2018/06/cat_stuck_in_	11.09.2019	*Felis catus*

hellmans_jar_brings_out_the_best_in_r.html		
https://www.facebook.com/permalink.php?story_fbid=10155481056179583&id=167122824582&substory_index=0	12.09.2019	*Mephitis mephitis*
https://www.news9.com/category/169390/archive-101209-monday	12.09.2019	*Mephitis mephitis*
https://www.islandpacket.com/news/local/news-columns-blogs/untamed-lowcountry/article198110654.html	12.09.2019	*Odocoileus* sp.
http://www.prairiestateoutdoors.com/pso/article/tale_of_the_buckethead_buck	12.09.2019	*Odocoileus* sp.
https://www.thestar.com/news/gta/2010/07/21/bears_head_stuck_in_jar_for_two_weeks_tale_of_a_rescue_effort.html	12.09.2019	*Ursus americanus*
https://www.youtube.com/watch?time_continue=22&v=vARVtdrhZFc	12.09.2019	*Procyon lotor*
https://www.youtube.com/watch?v=qHXRZhJAltg	12.09.2019	*Canis lupus familiaris*
https://www.animalrahat.com/hunger-led-ameesha-into-a-death-trap/	12.09.2019	*Canis lupus familiaris*
https://newsofmonth.com/video/the-dog-rescued-from-the-head-stuck-in-the-plastic-bottle/	12.09.2019	*Canis lupus familiaris*
https://www.dailymail.co.uk/news/article-2154934/Facebook-rescue-Stray-dog-head-stuck-container-saved-flashmob-rescue-party.html	12.09.2019	*Canis lupus familiaris*
https://sowegalive.com/2016/01/05/problems-with-litter-plays-havoc-with-dog/	12.09.2019	*Canis lupus familiaris*
https://www.youtube.com/watch?v=NyIVgfTr-sE	12.09.2019	*Canis lupus familiaris*
https://www.youtube.com/watch?v=G4EH5hSxSk4	12.09.2019	*Canis lupus familiaris*
http://www.mangaloretoday.com/main/Stray-dog-with-plastic-bottle-stuck-on-head-finally-rescued.html	12.09.2019	*Canis lupus familiaris*
https://wpde.com/news/local/dog-saved-after-getting-head-stuck-in-plastic-container-in-marion-county	12.09.2019	*Canis lupus familiaris*
https://www.straitstimes.com/asia/se-asia/malaysia-rangers-rescue-dog-stuck-in-plastic-container	12.09.2019	*Canis lupus familiaris*
https://www.youtube.com/watch?v=yp4Ll_hLLQs	12.09.2019	*Canis lupus familiaris*
https://timesofindia.indiatimes.com/city/pune/dog-with-head-stuck-in-jar-for-21-days-saved/articleshow/60706718.cms	12.09.2019	*Canis lupus familiaris*
https://abc7.com/pets/dog-rescued-after-getting-head-stuck-in-plastic-jar/315711/	12.09.2019	*Canis lupus familiaris*
https://www.newsflare.com/video/259904/animals/kind-volunteer-rescues-stray-dog-with-plastic-jar-stuck-on-its-head	12.09.2019	*Canis lupus familiaris*

https://www.newsflare.com/video/270458/animals/street-dog-that-got-its-head-stuck-inside-a-plastic-jar-rescued-after-hours-long-operation-in-india	12.09.2019	*Canis lupus familiaris*
https://indianexpress.com/article/trending/trending-in-india/tweeple-laud-bengaluru-cops-who-rescued-a-stray-dog-head-stuck-in-a-water-pot-4955622/	12.09.2019	*Canis lupus familiaris*
https://www.youtube.com/watch?v=xA_0G0ACOx4	12.09.2019	*Canis lupus familiaris*
https://www.newsflare.com/video/282213/animals/feral-indian-dog-rescued-after-getting-its-head-stuck-in-jar	12.09.2019	*Canis lupus familiaris*
https://mumbaimirror.indiatimes.com/mumbai/other/dog-whose-head-was-stuck-in-a-plastic-jar-freed-after-a-week/articleshow/66289578.cms	12.09.2019	*Canis lupus familiaris*
https://www.upi.com/Odd_News/2018/11/26/Man-rescues-puppy-with-jar-stuck-on-its-head/8961543259607/	12.09.2019	*Canis lupus familiaris*
https://www.puppyleaks.com/dog-head-stuck-jar-rescued/	12.09.2019	*Canis lupus familiaris*
https://www.gazettelive.co.uk/news/teesside-news/hungry-cat-rescued-rspca-after-8531690	13.09.2019	*Felis catus*
https://www.catster.com/lifestyle/cat-with-head-stuck-in-can-finds-her-way-to-a-shelter	13.09.2019	*Felis catus*
https://www.animal-zone.org/news/2019/6/8/the-danger-of-empty-cans	13.09.2019	*Felis catus*
https://lindsaywildlife.wordpress.com/2011/01/07/safely-dispose-of-trash/	13.09.2019	*Sciurus niger*
https://www.mirror.co.uk/news/uk-news/watch-heartwarming-moment-baby-fox-4269089	13.09.2019	*Vulpes vulpes*
http://www.fox13news.com/news/local-news/74030816-gallery	13.09.2019	*Procyon lotor*
https://www.youtube.com/watch?v=EsoCHjNOE0k	13.09.2019	*Mephitis mephitis*
https://www.mirror.co.uk/news/world-news/hungry-kitten-rescued-smashing-glass-8741109	13.09.2019	*Felis catus*
https://news.rspca.org.uk/2017/10/30/what-a-cat-astrophie-puss-is-rescued-after-getting-tin-can-stuck-on-head/	13.09.2019	*Felis catus*
https://www.youtube.com/watch?v=lOwr_i7QkCI	13.09.2019	*Felis catus*
https://www.lifewithcats.tv/2014/10/22/foraging-cat-with-head-stuck-in-cat-food-can-gets-help/	13.09.2019	*Felis catus*
https://www.expressandstar.com/news/local-hubs/walsall/2019/03/28/watch-cat-saved-after-getting-head-stuck-in-jar/	13.09.2019	*Felis catus*
https://petcatmiaw.blogspot.com/2017/12/eunos-community-cat-had-head-stuck-in.html	13.09.2019	*Felis catus*
https://www.wideopenpets.com/cat-rescued-spending-week-head-stuck-jar-mayo/#fb-comments-wrapper	13.09.2019	*Felis catus*

https://www.dailymail.co.uk/news/article-4593640/Hungry-cat-escapes-getting-head-stuck-soup-can.html	13.09.2019	*Felis catus*
https://www.caymancompass.com/2017/07/11/cat-stuck-in-bottle/	13.09.2019	*Felis catus*
https://stv.tv/news/west-central/1436436-waste-warning-after-cat-gets-head-stuck-in-tin-can/	13.09.2019	*Felis catus*
https://metro.co.uk/2016/05/27/cat-gets-head-stuck-in-jar-has-to-be-rescued-by-rspca-5908902/	13.09.2019	*Felis catus*
https://patch.com/new-york/massapequa/massapequa-cat-found-head-stuck-soup-can-needs-home-0	13.09.2019	*Felis catus*
https://www.irishmirror.ie/news/irish-news/cat-recovering-after-spending-week-9409383	13.09.2019	*Felis catus*
https://abc30.com/pets-animals/video-fresno-firefighters-help-cat-with-head-stuck-in-can/5156880/	13.09.2019	*Felis catus*
https://news.rspca.org.uk/2017/06/28/what-a-cat-astrophe-puss-rescued-by-rspca-after-getting-tin-can-stuck-on-head/	13.09.2019	*Felis catus*
https://stories.swns.com/news/cat-rescued-firefighters-head-stuck-tin-food-38980/	13.09.2019	*Felis catus*
http://pugetsoundblogs.com/kitsap-crime/2012/10/26/kitsap-deputy-saves-cat-with-tuna-can-stuck-to-her-head-with-photo/	13.09.2019	*Felis catus*
https://www.dailypicksandflicks.com/2017/01/18/man-helps-free-cat-with-head-stuck-in-can/	13.09.2019	*Felis catus*
https://viraltab.news/hungry-stray-cat-has-tin-can-stuck-firmly-on-its-head/	13.09.2019	*Felis catus*
https://eu.thedailyjournal.com/story/news/2016/02/09/millville-stray-escapes-sticky-situation/80078506/	13.09.2019	*Felis catus*
https://www.bbc.com/news/av/uk-england-lincolnshire-35731991/curious-cat-cut-free-from-dog-food-tin-in-lincolnshire	13.09.2019	*Felis catus*
https://www.youtube.com/watch?v=u_ruqLgruIk	13.09.2019	*Felis catus*
https://twitter.com/karlii_mcgovern/status/760833305151434752	13.09.2019	*Felis catus*
http://www.animalalliancenyc.org/wordpress/2016/09/thanks-supporters-pinky-cat-safe/	13.09.2019	*Felis catus*
https://www.facebook.com/rossvillefirepolice/posts/1287729171362133	13.09.2019	*Felis catus*
https://knews.kathimerini.com.cy/en/news/cat-has-run-in-with-police-in-larnaca	13.09.2019	*Felis catus*
https://kittentoob.com/stray-kitten-with-his-head-stuck-in-a-garbage-can-gets-a-happy-ending/	13.09.2019	*Felis catus*
https://www.animalrahat.com/littering-hurts-animals/	13.09.2019	*Felis catus*
https://www.bbc.com/news/uk-england-coventry-warwickshire-34779998	13.09.2019	*Felis catus*
https://animalchannel.co/rescuing-cat-stuck-can/	13.09.2019	*Felis catus*

https://www.clevescene.com/scene-and-heard/archives/2014/02/21/photo-canton-community-helps-rescue-cat-with-plastic-bottle-stuck-on-its-head	13.09.2019	*Felis catus*
http://www.bocian.org.pl/artykuly/niebezpieczne-butelki	15.09.2019	*Anoplotrupes stercorosus*
http://www.gdos.gov.pl/plastikowe-butelki-smiertelna-pulapka-dla-zwierzat	15.09.2019	*Anoplotrupes stercorosus, Carabus glabratus, Carabus hortensis, Nicrophorus vespillo*
http://www.uwm.edu.pl/czachor/wspomnienia_pliki/2008/2008.htm	15.09.2019	*Anoplotrupes stercorosus, Carabus glabratus*
https://www.facebook.com/photo.php?fbid=2643114255698657&set=a.233050930038347&type=3&theater	15.09.2019	*Trypocornis vernalis*
http://www.przyroda.mazury.pl/index.php?page=nature&id=233	15.09.2019	*Carabidae*
https://www.facebook.com/lasbemowski/photos/a.1516252878425684/2712171162167177/?type=3&theater	15.09.2019	*Anoplotrupes stercorosus, Nicrophorus vespillo*
https://twitter.com/fsologists_ak/status/644244551838793728/photo/1	16.09.2019	*Ursus americanus*
https://icelandmag.is/article/a-cow-gets-trouble-after-sticking-its-head-a-bucket	16.09.2019	*Bos taurus taurus*
https://www.facebook.com/photo.php?fbid=10207633232099452&set=p.10207633232099452&type=3&theater	16.09.2019	*Mephitis mephitis*
https://www.facebook.com/photo.php?fbid=10206482465113038&set=o.291872184313090&type=3&theater	16.09.2019	*Mephitis mephitis*
https://www.youtube.com/watch?v=R1D79uYQaLo&feature=youtu.be&fbclid=IwAR1MmOJAzyw_yEZ5SrzmB-tpBoGYTYYueM75tirk4FRmO43ZHlVAOkI1sDA	16.09.2019	*Mephitis mephitis*
https://www.mlive.com/news/detroit/2015/08/watch_brave_michigan_cop_pull.html	16.09.2019	*Mephitis mephitis*
https://www.youtube.com/watch?v=oOg70Eicqy8&feature=youtu.be&fbclid=IwAR12SHfStSaXKbPBnyMeOX1ZP1wR3d3xoCJTC-8Q83OakgMyx1zz02bU7VY	16.09.2019	*Mephitis mephitis*
https://www.youtube.com/watch?v=9uLaXBPvxSs&fbclid=IwAR1S3uh4y9lHwIt8ukzKvWUWvg524mr54ntGlvrzul23g0pXBLc06NEqXt8	16.09.2019	*Mephitis mephitis*
https://www.youtube.com/watch?v=s9_gxYFG_1Y&f	16.09.2019	*Mephitis mephitis*

eature=youtu.be&fbclid=IwAR0PGk5eZCAAMcKwpJzfQTf8SGrgb3TpxmMmqBosMhozNqxtRqSfSEJL_m8		
https://www.gettyimages.com/detail/video/skunk-wandering-around-park-with-head-stuck-in-yoplait-news-footage/149851769?fbclid=IwAR2kKN1WkME7Mrv4bZLBfcD-qizGUoP0nSgxHnCv9tXJUV18u09soJPBzBw	16.09.2019	*Mephitis mephitis*
https://www.huffpost.com/entry/squirrel-yogurt-cup-yoplait_n_57b1dd1fe4b0718404120302?fbclid=IwAR32DbjQ-H85zemUxl0AYqLpGbayMEJlo_AJRjRgJKz7fH9mosUfuIlTQPw	16.09.2019	*Sciuridae*
https://www.youtube.com/watch?v=BOnXjfESAgw&feature=youtu.be&fbclid=IwAR2wGC9l3R8JKHAuOX20g6LERi9IESWV-aTwhoQhVMFhfwHysSKAfciyWMA	16.09.2019	*Sciurus carolinensis*
https://www.youtube.com/watch?v=sSAt8lgQe_M&feature=youtu.be&fbclid=IwAR1u3zsnxhSVCfHpNEezLilOrn868HiWslKbMyj9b2MxGDFVdavfgsTvxKM	16.09.2019	*Sciuridae*
https://www.youtube.com/watch?v=muJ9YMDTvwA&feature=youtu.be&fbclid=IwAR08QnU3EEoKOJGDnGPBwd3NBL2hD10wUO9hJdBSdIWcG8Zd_00GFzes2Bw	16.09.2019	*Sciurus carolinensis*
https://www.youtube.com/watch?v=3eLaAI3Mnag&feature=youtu.be&fbclid=IwAR3YgTLmyFOM_Jp2jbEswT5AxkqMk9s84iICAQVOjeT7_RXwCrMaVSKwDv4	16.09.2019	*Sciurus niger*
https://www.youtube.com/watch?v=PXccGB9AkBo&feature=youtu.be&fbclid=IwAR0l5Rqra6w99cqb8jxwBRcntRoiXAps1XNP1xLGfYqcf4-11-rwaMZx8hY	16.09.2019	*Sciuridae*
https://www.youtube.com/watch?v=psKekF1ve3Y&feature=youtu.be&fbclid=IwAR0NM7EAdHOzRojUh_ywpJX78e1mSmHYNUDxmacndmJPDhbtthRoQIWkxMQ	16.09.2019	*Sciurus carolinensis*
https://www.cbc.ca/news/canada/sudbury/bear-gets-head-stuck-in-plastic-jar-in-greater-sudbury-1.2616233	16.09.2019	*Ursus americanus*
https://www.dailymail.co.uk/news/article-4179576/You-kidding-Silly-goats-head-stuck-jar.html	16.09.2019	*Capra hircus*
https://www.dailymail.co.uk/news/article-3609400/Now-looks-like-tight-jam-Young-fox-cub-gets-head-stuck-jam-car-freed-RSPCA.html	16.09.2019	*Vulpes vulpes*
https://www.foxnews.com/science/new-york-firefighters-free-raccoon-with-head-stuck-in-mayo-jar	16.09.2019	*Procyon lotor*
https://www.dailymail.co.uk/news/article-	16.09.2019	*Vulpes vulpes*

1181224/Pictured-The-cub-got-head-stuck-outfoxed-jar.html		
https://www.dailymail.co.uk/news/article-2508765/Jam-jar-Adorable-baby-raccoon-rescued-getting-head-stuck-jar-video.html	16.09.2019	*Procyon lotor*
https://eu.usatoday.com/story/news/nation-now/2015/11/06/raccoon-rescued-after-head-stuck-jar-missouri/75299510/	16.09.2019	*Procyon lotor*
https://www.youtube.com/watch?v=mlKla4VjnkQ	17.09.2019	*Canis lupus familiaris*
https://imgur.com/gallery/SpVoA/comment/9093678	17.09.2019	*Felis catus*
https://renkum.nieuws.nl/nieuws/2138/egeltje-vast-in-weggegooide-beker/	17.09.2019	*Erinaceus europaeus*
https://www.sunshinecoastdaily.com.au/news/beach-clean-up-aims-to-stop-rubbish-entering-our-o/3169501/	17.09.2019	*Lutjanus* sp.
https://www.youtube.com/watch?v=XLtYLZiPaeY	17.09.2019	*Varanus komodoensis*
https://www.newsflare.com/video/312534/animals/poor-monitor-lizard-stumbles-along-road-with-snout-stuck-in-discarded-tin-in-thailand	17.09.2019	*Varanus salvator*
https://www.gettyimages.com/detail/news-photo/tin-can-sits-stuck-on-the-bill-of-a-red-crowned-crane-news-photo/139231846	17.09.2019	*Grus japonensis*
https://news.yahoo.com/raccoon-rescued-getting-face-stuck-165856260.html?guccounter=1&guce_referrer=aHR0cHM6Ly93d3cuZ29vZ2xlLmNvbS8&guce_referrer_sig=AQAAAMaytv0QpsFaWzeM_lAWev_YAyrN355XJ51_y6GzEYroyRGWdQ9u2uryT6EiFaNxLyib0Fo5EA8paNcOZEkskqscCs56COQQnu-N28Aw957nhZBodxgGmbBLrjO1a_iPtDHf9P_00890eBBKsic2-vYTFsEKM80d6SNrIAvTABhm	17.09.2019	*Procyon lotor*
https://metro.co.uk/2019/07/21/raccoons-hand-swells-four-times-size-getting-stuck-can-10435211/	17.09.2019	*Procyon lotor*
https://animalsgetout.wordpress.com/2015/10/01/226-600-5597-raccoon-s-get-out/animalsgetout-226-600-5597-raccoon-with-can-stuck-on-its-head/	17.09.2019	*Procyon lotor*
https://97x.com/police-officers-save-groundhog-with-head-stuck-in-tin-can/	17.09.2019	*Marmota monax*
https://citizen.co.za/news/1436523/likkewaan-rescued-after-being-stuck-in-a-tin-in-fast-flowing-river/	17.09.2019	*Varanus niloticus*
http://www.whitneyzone.com/wz/ubbthreads.php/topics/52119/Protect_your_trash_and_belongi	17.09.2019	*Sciuridae*
https://www.westerntelegraph.co.uk/news/14146145.cat-with-head-trapped-in-tin-can-rescued-by-electrician-from-pembrokeshire-shed/	17.09.2019	*Felis catus*

https://www.catster.com/the-scoop/cat-head-stuck-in-can-rescued	17.09.2019	*Felis catus*
https://www.avnblogfeed.com/police-video-action/police-rescue-cat-with-head-stuck-in-ravioli-can/	17.09.2019	*Felis catus*
https://thebestcatpage.com/2016/06/20/cat-walks-around-town-head-stuck-jar-days/	17.09.2019	*Felis catus*
https://ladyfreethinker.org/animal-rescue-helps-mischievous-raccoon-who-got-his-head-jammed-in-a-can/	17.09.2019	*Procyon lotor*
https://www.baytoday.ca/local-news/main-st-break-in-saves-kittens-696567	24.09.2019	*Felis catus*
https://www.dailymail.co.uk/news/article-2848816/Houston-area-family-rescues-dog-jug-head.html	24.09.2019	*Canis lupus familiaris*
https://www.lifewithdogs.tv/2013/09/animal-rescuers-save-dog-with-head-jammed-inside-plastic-container/	24.09.2019	*Canis lupus familiaris*
https://stock.adobe.com/ee/editorial/stray-dog-with-head-stuck-in-plastic-container-in-bangkok/147777306	24.09.2019	*Canis lupus familiaris*
https://blog.nature.org/science/2018/10/24/three-lizards-in-a-beer-can/	24.09.2019	*Elgaria multicarinata*
https://gephardtdaily.com/national-international/trapped-lizard-rescued-from-discarded-red-bull-can/	24.09.2019	Squamata
https://www.communitynews.com.au/kalamunda-reporter/news/swan-view-resident-hits-out-at-litterbugs-racehorse-goanna-dies-empty-can/	24.09.2019	*Varanus gouldii*
https://ananova.news/rare-species-die-inside-beer-bottle-dumped-in-desert/	24.09.2019	*Entomochilus horatii*, *Brachistostermus* sp., *Liolaemus nigromaculatus*
https://drive.google.com/open?id=1H6HtmeDM-luFToNShz4KQ6TmYPOyQ8xW	29.09.2019	Gastropoda
https://www.facebook.com/photo.php?fbid=889881771382671&set=p.889881771382671&type=3&theater	29.09.2019	*Micromys minutus*
https://www.facebook.com/IrishWildlifeTrust/photos/a.286536755103/10161869319620104/?type=3&theater	29.09.2019	*Vulpes vulpes*
https://www.littlehamptongazette.co.uk/news/people/littlehampton-fox-gets-its-head-stuck-in-plastic-container-1-8931643	12.09.2019	*Vulpes vulpes*
https://pets.ettoday.net/news/1154702	20.10.2019	*Felis catus*
http://happytify.cc/article/592517	20.10.2019	*Felis catus*
https://pets.ettoday.net/news/448267	20.10.2019	*Canis lupus familiaris*

https://new.qq.com/omn/20180802/20180802A0XZZF.html	20.10.2019	*Canis lupus familiaris*
http://m.taihainet.com/news/fujian/szjj/2008-02-23/222071.html	20.10.2019	*Paguma larvata*
https://xw.qq.com/partner/hwbrowser/20190709A0CQOG/20190709A0CQOG00?ADTAG=hwb&pgv_ref=hwb&appid=hwbrowser&ctype=news	20.10.2019	*Felis catus*
https://baijiahao.baidu.com/s?id=1594276682972795479	20.10.2019	*Felis catus*
http://www.cqsccw.com/xinwen/wenzhang/1014.html	20.10.2019	*Canis lupus familiaris*
https://baijiahao.baidu.com/s?id=1630968653684177487	20.10.2019	*Felis catus*
http://www.sohu.com/a/283532424_564559	20.10.2019	*Canis lupus familiaris*
https://read01.com/Ny43G8K.html#.XayHyH_grIV	20.10.2019	Serpentes
https://www.google.com/imgres?imgurl=http%3A%2F%2Fy3.ifengimg.com%2Fcmpp%2F2014%2F09%2F04%2F11%2Fca6c4ef1-1f3c-4be5-8f38-39b0e9aa1437.jpg&imgrefurl=http%3A%2F%2Fnews.ifeng.com%2Fa%2F20140904%2F41861961_0.shtml&docid=wrZPrjEoui0HHM&tbnid=COTC-FOG4FR4zM%3A&vet=10ahUKEwjgl7C1j6vlAhUyyKYKHQgFCYQQMwhHKAUwBQ..i&w=485&h=647&itg=1&client=firefox-b-d&bih=804&biw=1696&q=%E5%8D%A1%E5%9C%A8%E7%BD%90%E5%AD%90%E8%A3%A1%E7%9A%84%E8%9B%87&ved=0ahUKEwjgl7C1j6vlAhUyyKYKHQgFCYQQMwhHKAUwBQ&iact=mrc&uact=8	20.10.2019	Serpentes
https://lalluram.com/national-news-cobra-snake-rescue-beer-bottel/	20.10.2019	*Naja naja*
https://www.schnauzi.com/perro-callejero-cabeza-atascada-bote-plastico-encontrado/	20.10.2019	*Canis lupus familiaris*
https://www.ahorasalta.com.ar/noticias/nacionales-9/video-salvo-a-un-zorro-de-morir-asfixiado-tenia-en-su-cabeza-dos-cajas-de-tetra-brik-8881	20.10.2019	*Lycalopex griseus*
https://espanol.upi.com/Curiosidades/2017/02/22/Un-hombre-de-Idaho-libera-a-un-ciervo-que-tena-la-cabeza-atrapada-en-un-barril/8361487784630/	20.10.2019	*Odocoileus* sp.
https://www.dailymail.co.uk/news/article-2232303/Dog-coffee-neck-makes-recovery-surgeries-Dallas-Texas.html	20.10.2019	*Canis lupus familiaris*
https://www.20minutos.es/fotos/actualidad/24-horas-en-fotos-1973/1/	20.10.2019	*Felis catus, Murinae*
http://kikomar.blogspot.com/2012/10/el-zorrillo-enfrascado.html	20.10.2019	*Mephitis macroura*

https://www.istockphoto.com/au/photo/dead-trapped-green-grass-snake-in-a-rusty-metal-can-gm466973343-33788856	20.10.2019	*Thamnophis* sp.
https://www.humanegardener.com/skunks-better-pesticides/	20.10.2019	Mephitidae
http://nairobiwire.com/2013/05/mombasa-residents-refuse-to-save-cat.html	20.10.2019	*Felis catus*
https://www.alamyimages.fr/photo-image-souris-domestique-mus-domesticus-pris-dans-une-bouteille-en-plastique-77484664.html	19.09.2019	*Mus musculus*
https://www.vosgesmatin.fr/edition-d-epinal/2017/08/18/un-chat-la-tete-coincee-dans-un-pot-de-pate-a-tartiner-et-personne-n-arrive-a-l-attraper	19.09.2019	*Felis catus*
https://www.gentside.com/ours/un-ours-s-est-coince-la-tete-dans-un-pot-en-plastique-en-images_art14643.html	19.09.2019	*Ursus arctos*
https://fr.metrotime.be/2017/02/14/actualite/monde/video-policier-neo-zelandais-sauve-herisson-coince-pot-de-yaourt/	19.09.2019	*Erinaceus europaeus*
https://fr.lovely-dogs.com/1039-dog-head-in-jar	19.09.2019	*Canis lupus familiaris*
https://www.dailymotion.com/video/x17l4x2	19.09.2019	*Canis lupus familiaris*
https://fr.dreamstime.com/clips-vid%C3%A9os-mouche-coll%C3%A9-r%C3%A9sidu-d-alcool-%C3%A0-l-int%C3%A9rieur-bouteille-essayant-ressource-se-lib%C3%A9rer-video63933646	19.09.2019	Insecta
https://www.koreus.com/video/rat-actimel.html	19.09.2019	*Rattus norvegicus*
https://www.tuasvu.com/un-chat-sauve-in-extremis-la-tete-coincee-dans-une-boite-de-conserve/	19.09.2019	*Felis catus*
http://wavepets.com/chien-avec-une-bouteille-coincee-sur-la-tete-pendant-des-jours-sauves/	19.09.2019	*Canis lupus familiaris*
https://www.youtube.com/watch?v=lIY6zVkQ-fY	19.09.2019	*Canis lupus familiaris*
http://www.madpsx.com/cabot-stray-sauve-apres-avoir-erre-pendant-des-jours-avec-la-tete-coincee-dans-un-bocal/	19.09.2019	*Canis lupus familiaris*
http://www.madpsx.com/chien-avec-la-tete-coincee-dans-un-bocal-sauve-par-les-prisonniers/	19.09.2019	*Canis lupus familiaris*
http://www.madpsx.com/minou-doux-avec-la-tete-coincee-dans-une-poubelle-sauve-par-michigan-humane-society/	19.09.2019	*Felis catus*
https://www.frasercoastchronicle.com.au/news/fraser-island-image-released-lizard-trapped-beer-c/3094586/	19.09.2019	*Bellatorias frerei*
https://www.icebreaker.com/fr-se/move-to-natural.html	19.09.2019	*Fish*
https://www.youtube.com/watch?v=-O15yr2gn8w	19.09.2019	Mugilidae

http://mes-photos-57400.over-blog.com/article-36795003.html	19.09.2019	Murinae
https://www.nationalgeographic.com/photography/your-shot/photos-of-the-week/2019/03/best-pictures-week-of-march-15-from-our-community/?cmpid=org=ngp::mc=social::src=facebook::cmp=editorial::add=fb20191023photo-resurfpotwsecondlook::rid=&sf222030361=1&fbclid=IwAR2O1wUhCUoEt2oh0eZjmzYtYvQpCwFAYhe_4Wmk1kbq_ek8xPseilrNmCw	23.10.2019	Malacostraca
https://wiadomosci.wp.pl/wyglodnialy-jez-utknal-w-kubku-po-jogurcie-strazacy-ruszyli-na-pomoc-6288539043882625a	30.10.2019	*Erinaceus europaeus*
https://metro.co.uk/2016/09/03/hedgehog-proves-why-you-shouldnt-always-lick-the-lid-of-life-6108108/	30.10.2019	*Erinaceus roumanicus*
https://www.mirror.co.uk/news/weird-news/video-hedgehog-gets-head-stuck-3391457	30.10.2019	*Erinaceus europaeus*
https://gloswielkopolski.pl/poznan-znalezli-kota-z-puszka-na-glowie/ar/437288	30.10.2019	*Felis catus*
https://www.o2.pl/artykul/tego-kota-szuka-cale-miasto-znalazl-sie-w-smiertelnej-pulapce-6227943285757569a	30.10.2019	*Felis catus*
https://www.tvp.info/37441614/lisek-wlozyl-glowe-w-sloik-i-klops-strazacy-uwolnili-bezradne-zwierze	30.20.2019	*Vulpes vulpes*
https://wiadomosci.wp.pl/puszcza-niepolomicka-mlody-lis-zaklinowal-sie-w-sloiku-potrzebowal-pomocy-6418743305259137a	30.20.2019	*Vulpes vulpes*
https://www.tvn24.pl/wroclaw,44/swidnica-sarna-z-plastikowa-butelka-na-glowie-mieszkancy-na-tropie,848506.html	30.10.2019	*Capreolus capreolus*
https://www.eldia.es/tenerife/2019/10/19/lata-trampa-mortal/1017851.html	30.10.2019	*Pimelia ascendens*
https://www.critica.com.pa/insolitas/bomberos-utilizan-mandibula-de-la-vida-para-rescatar-oso-atrapado-en-una-lata-528307	30.10.2019	*Ursus americanus*
http://g1.globo.com/planeta-bizarro/noticia/2014/03/cobra-e-salva-apos-entalar-cabeca-em-lata-de-refrigerante-na-australia.html	30.10.2019	*Pseudechis porphyriacus*
https://www.youtube.com/watch?v=FPeB2RzDfcU	30.10.2019	*Felis catus*
https://www.schnauzi.com/wong-perro-salvado-cabeza-atrapada-bote-plastico/	30.10.2019	*Canis lupus familiaris*
http://www.press.lv/post/pozhalujsta-pokazhite-eto-svoim-detyam-rizhanin-spas-ezha-zastryavshego-v-musore/	30.10.2019	*Erinaceus roumanicus*
https://animalreader.ru/v-magnitogorske-spasli-zastryavshuyu-v-banke-sobaku.html	30.10.2019	*Canis lupus familiaris*
https://fishki.net/1540467-po-stopam-vinni-puha-16-	30.10.2019	*Felis catus*

zastrjavshih-zverja/gallery-2032515-4-ulichnaja-koshka-v-banke-iz-pod-supa-photo.html		
https://kh.vgorode.ua/news/sobytyia/252634-v-kharkove-spasly-schenka-kosmonavta-zastriavsheho-v-banke-s-makaronamy	30.10.2019	*Canis lupus familiaris*
https://yle.fi/uutiset/osasto/novosti/lebed_s_zastryavshyei_v_klyuve_pivnoi_bankoi_i_ptitsy_zaputavshiesya_v_leske_-_foto_kak_musor_kalechit_dikikh_zhivotnykh/9725789	30.10.2019	*Cygnus cygnus*
http://www.gig26.ru/news/proishestviya/nid-11516.html	30.10.2019	*Canis lupus familiaris*
https://sakhapress.ru/mobile/archives/247692	30.10.2019	*Canis lupus familiaris*
https://www.yaplakal.com/forum13/topic1062566.html	30.10.2019	*Lepus europaeus*
https://news.rambler.ru/other/40102314-v-kurskoy-oblasti-neravnodushnye-lyudi-spasli-zastryavshego-v-butylke-lisenka/?updated	30.10.2019	*Vulpes vulpes*
https://www.theboltonnews.co.uk/news/14093077.hedgehog-with-head-stuck-in-mayonnaise-bottle-rescued-by-firefighters/	30.10.2019	*Erinaceus europaeus*
https://www.youtube.com/watch?v=qs0AIxhvY_Y	30.10.2019	*Canis lupus familiaris*
https://www.valleynewslive.com/home/headlines/Bear-Stuck-in-Cream-Can-351134111.html	30.10.2019	*Ursus americanus*
https://wrex.com/category/2019/08/16/squirrel-with-coke-can-stuck-on-head-rescued-by-humane-society-in-wisconsin/	30.10.2019	*Sciuridae*
https://www.google.com/imgres?imgurl=https://media.gettyimages.com/photos/dont-litter-picture-id575713453?b%3D1%26k%3D6%26m%3D575713453%26s%3D612x612%26h%3D9Mq3IOxYDs2SKErfBYLxvG_VRZc5eiFPBZeWIuJs18w%3D&imgrefurl=http://www.galloimages.co.za/image/mouse-is-seen-stuck-inside-beer/575713453/2&docid=_tRVIMleIptBMM&tbnid=bpMd_68XHFbBLM:&vet=1&w=612&h=510&itg=1&source=sh/x/im	30.10.2019	*Murinae*
https://rusturkey.com/post/202756/v-antale-spasli-sobaku-zastryavshuyu-golovoj-v-butylke	30.10.2019	*Canis lupus familiaris*
http://www.tnrrealitycheck.com/trappedinjar.asp	30.10.2019	*Felis catus*
https://don24.ru/rubric/proisshestviya/lovushka-iz-butylki-rostovchane-vozmuscheny-deystviya-zhivoderov.html	30.10.2019	*Canis lupus familiaris*
https://news.tut.by/culture/590843.html	30.10.2019	*Felis catus*
https://fishki.net/1784888-v-novoshahtinske-spasli-	30.10.2019	*Canis lupus*

wenka-u-kotorogo-golova-zastrjala-v-plafone.html		*familiaris*
https://www.1obl.ru/news/o-lyudyakh/ne-spasaetsya-on-okhotnik-pytalsya-toporom-razbit-plastikovuyu-banku-na-golove-enota/	30.10.2019	*Nyctereutes procyonoides*
http://usolie-citi.ru/news/5624-usolskie-policeyskie-spasli-sobaku-golova-kotoroy-zastryala-v-banke.html	30.10.2019	*Canis lupus familiaris*
https://chehov-vid.ru/news/society/18858/v-pushchino-spasli-sobaku-kotoraya-pyat-dney-begala-v-banke-na-golove/	30.10.2019	*Canis lupus familiaris*
https://twitter.com/soft0paws/status/1101532400708960256	25.10.2019	*Felis catus*
https://www.youtube.com/watch?v=MBXXSFbjkVM	25.10.2019	*Canis lupus familiaris*
https://www.youtube.com/watch?v=_GXpkTQdlKg	25.10.2019	*Felis catus*
https://www.youtube.com/watch?v=Hlq04rmZwlE	25.10.2019	*Felis catus*
https://www.youtube.com/watch?v=_142BNz4_l8	25.10.2019	*Canis lupus familiaris*
https://noahsarkcares.blogspot.com/2016/08/bottle-with-his-head-stuck-in-container.html	25.10.2019	*Canis lupus familiaris*
https://www.newsflare.com/video/200517/animals/dog-rescued-after-getting-head-stuck-in-tin#	25.10.2019	*Canis lupus familiaris*
https://newyork.cbslocal.com/2019/09/28/long-island-raccoon-head-stuck-in-jar/	25.10.2019	*Procyon lotor*
https://www.newsflare.com/video/155778/animals/baby-snake-rescued-after-getting-stuck-in-fizzy-drink-can	25.10.2019	Serpentes
https://www.newsflare.com/video/168330/animals/stray-dog-rescued-after-two-weeks-of-head-stuck-in-plastic-jar	25.10.2019	*Canis lupus familiaris*
https://www.newsflare.com/video/244979/animals/rat-snake-struggles-to-free-its-head-stuck-in-beer-can	25.10.2019	*Pytas mucosa*
https://www.newsflare.com/video/277989/animals/furious-cobra-rescued-from-beer-can-in-east-india	25.10.2019	*Naja kaouthia*
https://www.newsflare.com/video/180649/animals/puppy-gets-her-head-stuck-in-a-peanut-can	25.10.2019	*Canis lupus familiaris*
https://www.rohoshyosondhane.com/animal-rescues-by-humans/	31.10.2019	*Canis lupus familiaris*
https://ont.by/news/medvezhonok-zastryal-golovoj-v-kyvshine-ego-spasli-policejskie	04.11.2019	*Ursus arctos*
https://www.youtube.com/watch?v=AgUsfuM2ndE	04.11.2019	*Varanus acanthurus*
https://www.picfair.com/pics/08256742-fire-salamander	05.11.2019	*Salamandra salamandra*
https://www.itv.com/news/central/2018-12-13/hedgehog-found-trapped-inside-tin-can/	06.11.2019	*Erinaceus europaeus*
http://1077thejewel.com/ontario-men-save-skunk-head-trapped-inside-pop-can/	06.11.2019	*Mephitis mephitis*
https://www.youtube.com/watch?v=Mq-2IbZPOfA	06.11.2019	*Mephitis mephitis*

https://www.nsnews.com/opinion/letters/time-to-raise-stink-over-skunk-traps-1.372431	06.11.2019	*Mephitis mephitis*
http://www.itchmo.com/officer-rescues-skunk-stuck-in-jar-with-bb-gun-3276	06.11.2019	*Mephitis mephitis*
https://www.youtube.com/watch?v=PbZicgAek60	06.11.2019	*Sciurus carolinensis*
https://www.youtube.com/watch?v=sKQJBt3IqCE	06.11.2019	*Sciuridae*
https://www.youtube.com/watch?v=kTxUS1Y8x7k	06.11.2019	*Erinaceus europaeus*
https://www.youtube.com/watch?v=oklUEn9p8gA	06.11.2019	*Erinaceus europaeus*
https://www.youtube.com/watch?v=bc37EW5nBfs	06.11.2019	*Erinaceus europaeus*
https://www.youtube.com/watch?v=E18shGWqOfY	06.11.2019	*Rattus* sp.
https://www.youtube.com/watch?v=ukjQ3216IjM	06.11.2019	*Rattus* sp.
https://www.youtube.com/watch?v=5lbnKNYEJEM	06.11.2019	*Sciurus carolinensis*
https://www.youtube.com/watch?v=Uc9vCH2Ybuc	06.11.2019	*Sciuridae*
https://www.youtube.com/watch?v=2uRsI5zeu3Y	06.11.2019	*Sciuridae*
https://www.youtube.com/watch?v=8Q_CNYLCy6k	06.11.2019	*Felis catus*
https://www.youtube.com/watch?v=lTSMkOl3zXk	07.11.2019	*Gallus gallus domesticus*
https://www.bbc.com/news/uk-england-somerset-48624427	07.11.2019	*Vulpes vulpes*
https://www.newsdogapp.com/en/article/5c53cd4612313a3e52de2949/?d=false	07.11.2019	*Austrelaps superbus*
https://www.news.com.au/lifestyle/real-life/wtf/in-todays-most-excellently-weird-story-guy-doing-charity-walk-survives-deadly-snake-bite-thanks-to-stormtrooper-suit/news-story/b8c9dee3a7981fc5e3cf709a124f182b	07.11.2019	*Pseudechis australis*
https://www.eastmojo.com/assam/2019/08/16/watch-how-a-monocled-cobra-got-its-head-stuck-in-empty-beer-can-2	07.11.2019	*Naja kaouthia*
https://www.dailymail.co.uk/news/article-2646900/Coke-extra-fizzz-Driver-stunned-SNAKE-stuck-inside-spotting-waving-road.html	07.11.2019	*Pseudechis colletti*
https://www.wildlife-rescue.org.au/snakes.html	07.11.2019	*Pseudechis porphyriacus*
http://www.wiresnr.org/Carersstories14.html	07.11.2019	*Pseudechis porphyriacus*
https://www.frasercoastchronicle.com.au/news/help-pepsi-the-snake-yes-i-can/1907238/	07.11.2019	*Pseudechis porphyriacus*
https://www.dailymail.co.uk/news/article-5588963/Venomous-tiger-snake-rescued-VB-Tasmanian-reptile-wrangler-recently-lost-finger.html	07.11.2019	*Notechis scutatus*
http://aliceonline.com.au/2010/03/24/dont-can-our-	07.11.2019	*Varanus gouldii*

lizards/		
http://thearkvet.com/wildlife-rescue/	07.11.2019	*Varanus scalaris*
https://www.facebook.com/northwoodanimalhospital/photos/a.406981605694/10156965550850695/?type=1&theater	07.11.2019	*Plestiodon laticeps*
https://www.youtube.com/watch?v=2_XZBfaAl9g	07.11.2019	Serpentes
https://www.youtube.com/watch?v=PSK5J3vtE6g	07.11.2019	*Ophiophagus hannah*
https://www.youtube.com/watch?v=gixb4w78kqQ	07.11.2019	*Varanus salvator*
https://www.youtube.com/watch?v=A5WhkeeJcLQ	07.11.2019	*Varanus nebulosus*
https://www.youtube.com/watch?v=78ndLOGH1rI	07.11.2019	*Poecile* sp.
https://www.youtube.com/watch?v=DHPVyhHszdg	08.11.2019	*Canis lupus familiaris*
https://5minutes.rtl.lu/actu/frontieres/a/1365284.html	08.11.2019	*Vulpes vulpes*
https://patch.com/connecticut/monroe/a-raccoon-gets-its-head-stuck-in-a-plastic-cup	08.11.2019	*Procyon lotor*
https://www.ngnews.ca/news/local/distressed-kitty-found-with-cup-stuck-on-head-78215/	08.11.2019	*Felis catus*
https://metro.co.uk/2018/12/04/turtle-rescued-plastic-cup-wrapped-around-neck-8209310/	08.11.2019	*Lepidochelys olivacea*
https://brewsterpolice.org/animal-control/animal-rescue/	08.11.2019	*Mephitis mephitis*
http://www.woaw.org.au/9to13/brushtail-possum-victim-litter-victim/	08.11.2019	*Trichosurus vulpecula*
https://twitter.com/hashtag/SkunkRemovalToronto?src=hash	08.11.2019	*Mephitis mephitis*
https://www.fieldandstream.com/photos/gallery/hunting/2009/06/how-rescue-black-bear-its-head-stuck-milk-jug/?dom=fas	08.11.2019	*Ursus americanus*
https://www.rivertowns.net/news/3739197-pierce-county-rescuers-made-awkward-situation-bear-able	08.11.2019	*Ursus americanus*
https://www.youtube.com/watch?v=0-KlNAvWqg0	08.11.2019	*Marmota monax*
https://www.youtube.com/watch?v=icapsMZ-kwg	07.11.2019	Fish
https://www.youtube.com/watch?v=oUjzx7UpZ5s	07.11.2019	*Canis lupus familiaris*
https://www.youtube.com/watch?v=lzpmnqSaejo	09.11.2019	Herpes*tidae*
https://www.newsflare.com/video/318513/cat-rescued-by-locals-after-getting-its-head-stuck-inside-plastic-jar#	09.11.2019	*Felis catus*
https://www.upi.com/Odd_News/2019/10/18/Florida-deputies-rescue-raccoon-with-plastic-bottle-stuck-on-head/2111571421727/	09.11.2019	*Procyon lotor*
https://hedgiesjoy.blogspot.com/2011/11/hidden-hedgehog-traps.html	09.11.2019	*Erinaceus europaeus*
http://jerseyhedgehogs.co.uk/page_1211894266703.html	09.11.2019	*Erinaceus europaeus*
https://www.youtube.com/watch?v=O0eFlwzLXjk	09.11.2019	*Mephitis mephitis*

https://www.youtube.com/watch?v=VDloGa2oLF0	09.11.2019	*Mephitis mephitis*
https://www.youtube.com/watch?v=cmx8gCkp3i0	09.11.2019	*Canis lupus familiaris*
https://www.youtube.com/watch?v=xOFaZFbZHa0	09.11.2019	*Canis lupus familiaris*
https://www.youtube.com/watch?v=v3fGzafQdsM	09.11.2019	*Felis catus*
https://www.youtube.com/watch?v=a6CoKJEprVk	09.11.2019	*Procyon lotor*
https://www.islandpacket.com/news/local/article231483438.html	21.11.2019	*Felis catus*
https://wyrk.com/pictures-kitten-gets-head-stuck-in-glass-bottle-in-amherst/	21.11.2019	*Felis catus*
https://imgur.com/gallery/gq7j7	21.11.2019	*Felis catus*
https://www.dailysabah.com/turkey/2019/08/01/turkish-firefighters-spend-10-days-to-rescue-cat-with-jar-stuck-on-neck	21.11.2019	*Felis catus*
https://abcnews4.com/news/local/good-samaritans-rescue-south-carolina-cat-with-head-stuck-in-jar-video	21.11.2019	*Felis catus*
https://www.cbs58.com/news/meet-chef-1-year-old-cat-rescued-after-being-found-with-head-stuck-in-can-of-spaghetti	21.11.2019	*Felis catus*
https://www.youtube.com/watch?v=r70xzfqyqlg	21.11.2019	*Felis catus*
https://www.youtube.com/watch?v=8y2lA_QNqb4	21.11.2019	*Felis catus*
https://www.youtube.com/watch?v=N-5kNi95FRM	21.11.2019	*Felis catus*
https://www.youtube.com/watch?v=pK5V_lM7Yj8	21.11.2019	*Felis catus*
https://www.youtube.com/watch?v=aVgg62b95UM	21.11.2019	*Felis catus*
https://www.youtube.com/watch?v=S5YDyeF0Cno	21.11.2019	*Felis catus*
https://www.youtube.com/watch?v=XCELX33L1t8	21.11.2019	*Felis catus*
https://www.youtube.com/watch?v=e8VujkLWoLE	21.11.2019	*Felis catus*
https://www.youtube.com/watch?v=CQ-_ANVYrHs	21.11.2019	*Urocyon cinereoargenteus*
https://www.youtube.com/watch?v=brCFoCfZtbg	21.11.2019	*Vulpes vulpes*
https://www.youtube.com/watch?v=zpFgvFH0JQg	21.11.2019	*Canis latrans*
https://www.youtube.com/watch?v=bN6cKrWR3w8	21.11.2019	*Canis latrans*
https://timesofindia.indiatimes.com/city/bhubaneswar/frantic-search-to-locate-stray-with-plastic-jar-stuck-on-head/articleshow/61790784.cms	27.11.2019	*Canis lupus familiaris*
https://www.youtube.com/watch?v=fKhpNZoc0OE	27.11.2019	*Canis lupus familiaris*
https://stock.adobe.com/ee/editorial/a-dog-with-a-plastic-container-stuck-in-its-head-walks-in-the-rebel-held-besieged-town-of-douma-eastern-damascus-suburb-of-ghouta/156894520	27.11.2019	*Canis lupus familiaris*
https://imgur.com/gallery/xtzTi	27.11.2019	*Canis lupus familiaris*
https://www.idaindia.org/dog-trapped-plastic-jar.htm	27.11.2019	*Canis lupus familiaris*
https://www.google.com/imgres?imgurl=https%3A%2	27.11.2019	*Canis lupus*

F%2Fwww.lifewithdogs.tv%2Fwp-content%2Fuploads%2F2013%2F09%2F130905-jar-head2.jpg&imgrefurl=https%3A%2F%2Fwww.lifewithdogs.tv%2Fpage%2F931%2F%3Fwt-stat%3Dtrue&docid=PyMBh1fjue3RqM&tbnid=mdcK0xMDU1DSVM%3A&vet=10ahUKEwiy_qGT1YrmAhUIx4sKHeUbC_4QMwhnKCYwJg..i&w=300&h=209&itg=1&bih=578&biw=1280&q=dog%20head%20in%20jar&ved=0ahUKEwiy_qGT1YrmAhUIx4sKHeUbC_4QMwhnKCYwJg&iact=mrc&uact=8		*familiaris*
https://www.berkeleyind.com/archives/dog-recovering-from-days-with-head-stuck-inside-jar/article_3d938486-c93b-576c-a612-9e73efe7adc0.html	27.11.2019	*Canis lupus familiaris*
https://www.animalrahat.com/latest-news/for-dogs-sake/	27.11.2019	*Canis lupus familiaris*
https://archive.shine.cn/nation/Hungry-stray-dog-saved-after-getting-its-head-stuck-in-jar-when-searching-for-food-inside/shdaily.shtml	27.11.2019	*Canis lupus familiaris*
https://yucatanexpatlife.com/stray-dog-in-a-peculiar-predicament-is-finally-captured-and-rescued/	27.11.2019	*Canis lupus familiaris*
https://english.mathrubhumi.com/news/offbeat/stray-dog-s-head-trapped-inside-pot-in-bid-to-catch-rat--1.3825983	27.11.2019	*Canis lupus familiaris*
https://english.mathrubhumi.com/news/offbeat/locals-rescue-stray-dog-whose-head-gets-stuck-inside-a-pot-1.2525696	27.11.2019	*Canis lupus familiaris*
http://www.taipeitimes.com/News/taiwan/archives/2012/01/31/2003524319	27.11.2019	*Canis lupus familiaris*
https://www.youtube.com/watch?v=r6d6sI7DNww	27.11.2019	*Canis lupus familiaris*
https://www.newsflare.com/video/324556/animals/rescuers-save-dog-in-india-with-head-trapped-in-plastic-container-for-ten-days	27.11.2019	*Canis lupus familiaris*
https://www.thegardenisland.com/2017/10/24/hawaii-news/dog-stuck-in-plastic-container-rescued/	27.11.2019	*Canis lupus familiaris*
https://www.fox23.com/news/trappers-looking-for-dog-with-jar-stuck-on-its-head-in-tulsa/1011034494	27.11.2019	*Canis lupus familiaris*
https://www.facebook.com/GoldCountryWildlifeRescue/posts/whos-looking-foxy-now-the-jar-head-fox-is-recovering-nicely-this-fox-has-gone-fr/1108930109263707/	27.11.2019	*Urocyon cinereoargenteus*
https://metro.co.uk/2016/06/17/tiny-fox-cub-dies-after-getting-stuck-in-plastic-bottle-5950224/	27.11.2019	*Vulpes vulpes*
https://www.facebook.com/tambylcy/videos/2437648549679968/?v=2437648549679968	28.11.2019	*Anoplotrupes stercorosus*
