# Supplementary material for: Online media reveals a global problem of discarded containers as deadly traps for animals
Source: Sci Rep. 2021 Jan 11;11:267. doi: 10.1038/s41598-020-79549-8 (PMC7801720; doi:10.1038/s41598-020-79549-8)
Supplement: Supplementary file 2 — Supplementary Table S2. [file 41598_2020_79549_MOESM2_ESM.pdf]

Online media reveals a global problem of discarded containers as deadly traps for animals

Krzysztof Kolenda, Monika Pawlik, Natalia Kuśmierek, Adrian Smolis, Marcin Kadej

Supplementary Table 2. List of countries in which animals trapped in discarded containers were observed by social media users. *Countries located across two continents were assigned to the one in which the incident occurred.

Continent	Country	Number of records
North America	USA	176
	Canada	19
	Mexico	2
	Costa Rica	1
	Belize	1
	Bermuda (United Kingdom)	1
	**total**	**200**
Europe	United Kingdom	46
	Poland	27
	Russia*	9
	Spain	6
	France	6
	Germany	3
	Ireland	3
	Iceland	2
	Netherlands	2
	Italy	1
	Ukraine	1
	Turkey*	1
	Switzerland	1
	Slovakia	1
	Latvia	1
	Greece	1
	Finland	1
	Cyprus	1
	Austria	1
	**total**	**114**
Asia	India	42
	Taiwan	8
	Russia*	7
	Thailand	6
	no data	6

	China	5
	Singapore	3
	Turkey*	2
	Malaysia	2
	South Korea	2
	Syria	1
	Nepal	1
	Kazakhstan	1
	Cambodia	1
	Japan	1
	Israel	1
	Indonesia	1
	Philippines	1
	Azerbaijan	1
	Saudi Arabia	1
	**total**	**93**
Australia and Oceania	Australia	42
	New Zealand	4
	**total**	**46**
Africa	RPA	2
	Algeria	1
	Kenya	1
	Mauritius	1
	Saint Helena Island (United Kingdom)	1
	**total**	**6**
South America	Argentina	1
	Chile	1
	Bonaire (The Netherlands)	1
	**total**	**3**
no data		41
	**total**	**41**
