# Supplementary material for: Online media reveals a global problem of discarded containers as deadly traps for animals
Source: Sci Rep. 2021 Jan 11;11:267. doi: 10.1038/s41598-020-79549-8 (PMC7801720; doi:10.1038/s41598-020-79549-8)
Supplement: Supplementary file 3 — Supplementary Table S3. [file 41598_2020_79549_MOESM3_ESM.pdf]

Online media reveals a global problem of discarded containers as deadly traps for animals

Krzysztof Kolenda, Monika Pawlik, Natalia Kuśmierek, Adrian Smolis, Marcin Kadej

Supplementary Table S3. Invertebrates that were found in discarded containers. nc – number of containers

**class**	**taxon**	**nc**
Arachnida	*Brachistostermus* sp.	1
Diplopoda	unidentified	1
Gastropoda	unidentified	1
Insecta	*Anoplotrupes stercorosus*	10
	Carabus glabratus	2
	Nicrophorus vespillo	2
	Carabidae	1
	Carabus coriaceus	1
	Carabus hortensis	1
	Coleoptera	1
	Entomochilus horatii	1
	Pimelia ascendens	1
	Trypocornis vernalis	1
	unidentified	1
Malacostraca	Paguroidea	1
	unidentified	1
