# Supplementary material for: Online media reveals a global problem of discarded containers as deadly traps for animals
Source: Sci Rep. 2021 Jan 11;11:267. doi: 10.1038/s41598-020-79549-8 (PMC7801720; doi:10.1038/s41598-020-79549-8)
Supplement: Supplementary file 4 — Supplementary Table S4. [file 41598_2020_79549_MOESM4_ESM.pdf]

Online media reveals a global problem of discarded containers as deadly traps for animals

Krzysztof Kolenda, Monika Pawlik, Natalia Kuśmierek, Adrian Smolis, Marcin Kadej

Supplementary Table S4. Vertebrates that were found in discarded containers and their IUCN Red List category (in alphabetical order). nc – number of containers. Mammals are additionally grouped by size category according to Lessa and Farina (1996): s – small, m – medium, l – large

class	taxon	nc	IUCN status
**fishes**	*Ictalurus furcatus*	1	LC
	Lutjanus sp.	1	-
	Mugilidae	1	-
	unidentified	2	-
**amphibians**	*Rhinella marina*	1	LC
	Salamandra salamandra	1	LC
**reptiles**	*Austrelaps* sp.	1	-
	Austrelaps superbus	5	LC
	Bellatorias frerei	1	LC
	Caretta caretta	1	VU
	Cheloniidae	1	-
	Coluber constrictor	1	LC
	Cyclodomorphus gerrardii	1	LC
	Dendroaspis polylepis	1	LC
	Elgaria multicarinata	1	LC
	Lacerta agilis	1	LC
	Lepidochelys olivacea	1	VU
	Liolaemus nigromaculatus	1	LC
	Masticophis lateralis	1	LC
	Naja kaouthia	2	LC
	Naja naja	3	-
	Naja sumatrana	1	LC
	Notechis scutatus	2	LC
	Ophiophagus hannah	1	VU
	Pituophis catenifer	2	LC
	Plestiodon laticeps	1	LC
	Plestiodon longirostris	1	CR
	Plestiodon sp.	1	-
	Podarcis muralis	1	LC
	Pseudechis australis	2	LC
	Pseudechis colletti	1	LC
	Pseudechis porphyriacus	11	LC
	Pseudonaja affinis	1	LC

	Pseudonaja textilis	1	LC
	Pytas mucosa	2	LC
	Squamata	3	-
	Serpentes	8	-
	Thamnophis sp.	1	-
	Varanus acanthurus	2	LC
	Varanus gouldii	3	LC
	Varanus komodoensis	1	VU
	Varanus nebulosus	1	-
	Varanus niloticus	1	-
	Varanus salvator	2	LC
	Varanus scalaris	1	LC
	Varanus tristis	2	LC
	Zootoca vivipara	1	LC
**birds**	*Anas platyrhynchos*	1	LC
	Cygnus cygnus	2	LC
	Gallus gallus domesticus	1	-
	Grus japonensis	1	EN
	Poecile sp.	1	-
**mammals**	*Apodemus agrarius* [s]	1	LC
	Apodemus flavicollis [s]	1	LC
	Apodemus sylvaticus [s]	1	LC
	Bos taurus taurus [l]	1	-
	Canis latrans [m]	10	LC
	Canis lupus familiaris [m]	79	-
	Canis lupus pallipes [m]	1	LC
	Capra hircus [m]	1	-
	Capreolus capreolus [m]	3	LC
	Dasyurus viverrinus [s]	1	EN
	Erinaceus europaeus [s]	19	LC
	Erinaceus roumanicus [s]	2	LC
	Felis catus [m]	88	-
	Herpestidae [m]	1	-
	Lepus europaeus [m]	1	LC
	Lycalopex griseus [m]	1	LC
	Macaca sp. [m]	1	-
	Marmota monax [m]	2	LC
	Meles meles [m]	1	LC
	Mephitidae [m]	1	-

Mephitis macroura [s]	1	LC
Mephitis mephitis [m]	39	LC
Micromys minutus [s]	1	LC
Murinae [s]	5	-
Mus musculus [s]	1	LC
Mustela nivalus [s]	1	LC
Nyctereutes procyonoides [m]	2	LC
Odocoileus sp. [m]	10	-
Odocoileus virginianus [m]	1	LC
Paguma larvata [m]	1	LC
Panthera pardus fusca [m]	1	VU
Phocidae [na]	1	-
Procyon lotor [m]	33	LC
Rattus norvegicus [s]	1	LC
Rattus sp. [s]	3	-
Sciuridae [s]	8	-
Sciurus carolinensis [s]	7	LC
Sciurus niger [s]	2	LC
Sciurus vulgaris [s]	2	LC
Sorex trowbridgii [s]	1	LC
Trichosurus vulpecula [m]	3	LC
Urocyon cinereoargenteus [m]	4	LC
Ursus americanus [l]	22	LC
Ursus arctos [l]	6	LC
Ursus maritimus [l]	1	VU
Vulpes vulpes [m]	23	LC
