# Supplementary material for: Online media reveals a global problem of discarded containers as deadly traps for animals
Source: Sci Rep. 2021 Jan 11;11:267. doi: 10.1038/s41598-020-79549-8 (PMC7801720; doi:10.1038/s41598-020-79549-8)
Supplement: Supplementary file 5 — Supplementary Table S5. [file 41598_2020_79549_MOESM5_ESM.pdf]

Online media reveals a global problem of discarded containers as deadly traps for animals

Krzysztof Kolenda, Monika Pawlik, Natalia Kuśmierek, Adrian Smolis, Marcin Kadej

Supplementary Table 5. Combination of key words and list of animal common names used during data collection

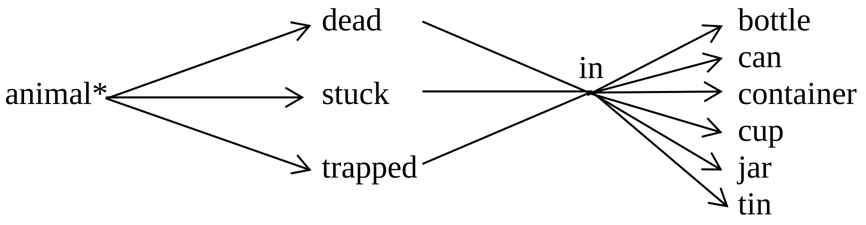

*also replaced by: arthropod, invertebrate, insect, bug, beetle, spider, arachnid, snail, worm, fish, amphibian, frog, toad, newt, salamander, reptile, snake, lizard, tortoise, turtle, bird, mammal, cat, coyote, dog, deer, rodent, marten, mice, shrew, skunk, squirrel, bear, hedgehog, vertebrate
